# Mucin degrader *Akkermansia muciniphila* accelerates intestinal stem cell-mediated epithelial development

**DOI:** 10.1080/19490976.2021.1892441

**Published:** 2021-03-08

**Authors:** Seungil Kim, Yun-Chan Shin, Tae-Young Kim, Yeji Kim, Yong-Soo Lee, Su-Hyun Lee, Mi-Na Kim, Eunju O, Kwang Soon Kim, Mi-Na Kweon

**Affiliations:** a Mucosal Immunology Laboratory, Department of Convergence Medicine, Asan Medical Center, University of Ulsan College of Medicine, Seoul, Republic of Korea; b Department of Laboratory Medicine, Asan Medical Center, University of Ulsan College of Medicine, Seoul, Republic of Korea; c Department of Life Sciences, Pohang University of Science and Technology, Pohang, Republic of Korea

**Keywords:** Gut microbiota, *akkermansia muciniphila*, intestinal stem cells, lgr5, mucin-degrading bacteria, g protein-coupled receptors, epithelial development, small intestine, organoids

## Abstract

Mucin-degrading bacteria are densely populated in the intestinal epithelium; however, their interaction with intestinal stem cells (ISCs) and their progeny have not been elucidated. To determine whether mucin-degrading bacteria play a role in gut homeostasis, mice were treated with *Akkermansia muciniphila*, a specialized species that degrades mucin. Administration of *A. muciniphila* for 4 weeks accelerated the proliferation of Lgr5^+^ ISCs and promoted the differentiation of Paneth cells and goblet cells in the small intestine (SI). We found similar effects of *A. muciniphila* in the colon. The levels of acetic and propionic acids were higher in the cecal contents of *A. muciniphila*-treated mice than in PBS-treated mice. SI organoids treated with cecal contents obtained from *A. muciniphila*-treated mice were larger and could be diminished by treatment with G protein-coupled receptor (Gpr) 41/43 antagonists. Pre-treatment of mice with *A. muciniphila* reduced gut damage caused by radiation and methotrexate. Further, a novel isotype of the *A. muciniphila* strain was isolated from heathy human feces that showed enhanced function in intestinal epithelial regeneration. These findings suggest that mucin-degrading bacteria (e.g., *A. muciniphila*) may play a crucial role in promoting ISC-mediated epithelial development and contribute to intestinal homeostasis maintenance.

## Introduction

Mammalian intestinal epithelial cells (IECs) have a rapid turnover rate and are replenished every 3–5 days.^1,2^ All types of IECs derive from intestinal stem cells (ISCs) that can generate either ISC daughters or proliferating progenitors called transit-amplifying (TA) cells.^3,4^ TA cells terminally differentiate into secretory cell lineages, such as Paneth, goblet, enteroendocrine, and enterocyte cells.^2^ Importantly, Paneth cells migrate down into the intestinal crypt bottom to produce ISC niche factors such as Wnt3, EGF, TGF-α, and Dll4.^5^

The Wnt signaling pathway plays an important role in promoting and driving the proliferative activity of ISCs and IEC differentiation.^6,7^ A recent study revealed that Wnt/β-catenin signaling supports gut homeostasis by maintaining self-renewal of Lgr5-expressing stem cells in the intestinal crypts.^8^ The Wnt ligand binds to the Frizzled and low-density lipoprotein receptor-related protein receptor. This leads to accumulation of β-catenin, the main mediator of the Wnt signal cascade in the gut,^9^ which translocates from the cytoplasm into the nucleus. Inside the nucleus, β-catenin binds with the transcription factor, TCF, to regulate genes involved in proliferation.^10,11^

Gut microbiota maintain gut homeostasis.^12,13^ Several studies have identified a link between dysbiosis and disease, such as inflammatory disorder, metabolic syndrome, and mental illness.^14–16^ Metabolites produced by gut microbiota are proposed to modulate host physiology.^14,17,18^ Short-chain fatty acids (SCFAs, e.g., acetic, propionic, butyric acids) are functional metabolites produced by bacterial fermentation of undigested complex carbohydrates.^19^ By binding to G protein-coupled receptors (Gpr) 41 and 43, SCFAs can affect host gut immunity and metabolism.^20,21^ In addition, we recently suggested that gut microbiota-derived lactate promotes IEC development in a Gpr81-dependent manner.^22^

*Akkermansia muciniphila* is a mucin-degrading bacterium and the sole genus of the phylum, Verrucomicobia, which is found in human stool.^23^ *A. muciniphila* represents approximately 1–3% of intestinal microbiota residing in the mucus layer near the IECs.^24,25^ Several studies have shown that the abundance of *A. muciniphila* is inversely correlated with various diseases, such as inflammatory bowel disease, diabetes, and obesity.^26–30^ Administration of *A. muciniphila* reduces weight gain and improves metabolic parameters, such as glucose metabolism.^27,29,31,32^ Metformin, an anti-diabetic agent, increases the abundance of *A. muciniphila* in the gut microbiota of obese mice under diet conditions.^33,34^ A recent study also demonstrated correlation between the clinical efficacy of immune checkpoint inhibitors and the relative abundance of *A. muciniphila*.^35^ These results indicate that *A. muciniphila* may have potential as a key next-generation microbe with a wide spectrum of therapeutic applications.

In this study, we investigated the potential role of *A. muciniphila* in IEC development. We confirmed that despite being a mucin-degrading bacterium, *A. muciniphila* paradoxically increased mucus production by promoting the differentiation of secretory IEC lineages. Administration of *A. muciniphila* enhanced ISC proliferation in a Gpr41/43-dependent manner and subsequently accelerated intestinal epithelial regeneration. Most importantly, *A. muciniphila* protected mice from severe gut damage caused by radiation and chemotherapy. Taken together, our findings suggest that *A. muciniphila* promotes IEC development and maintains gut homeostasis.

## Results

### Oral administration of A. muciniphila BAA-835 may promote epithelial differentiation in the SI

**Figure 1.**
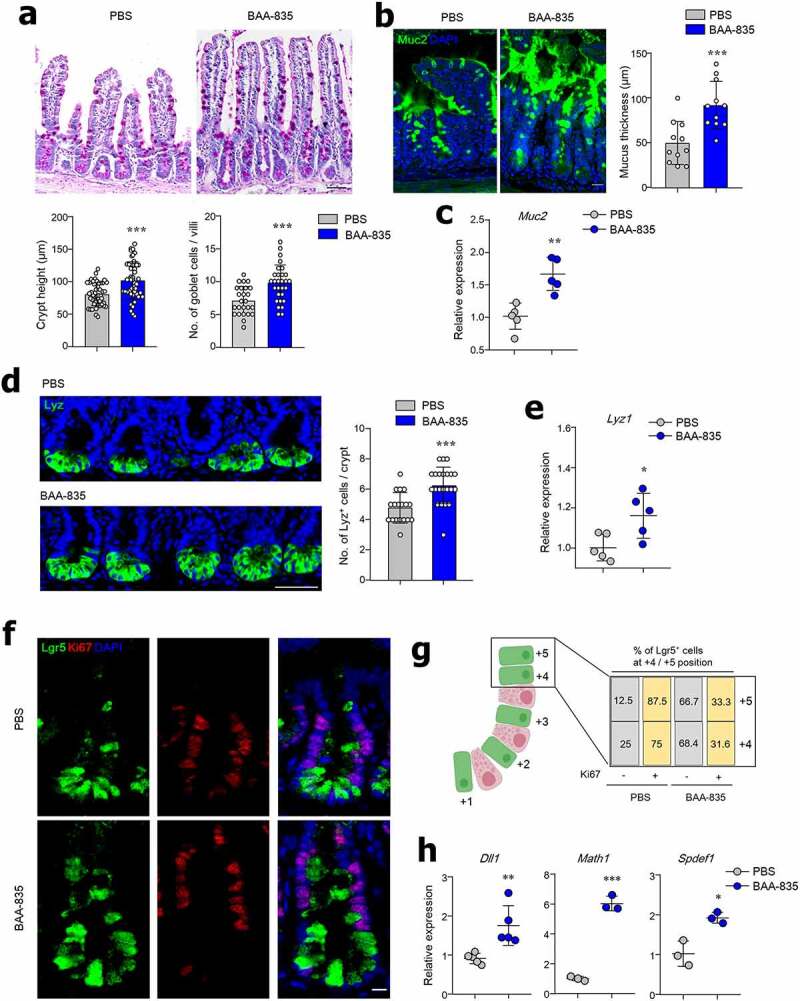
Mice treated with *A. muciniphila* BAA-835 had enhanced epithelial development and showed a thicker layer of mucus in the SI

To address whether mucin-degrading bacteria regulate IEC differentiation, BAA-835 was given orally to mice for 4 weeks. We first assessed the abundance of *A. muciniphila* in the SI contents of naïve B6 mice (Figure S1a). Colonization of BAA-835 in the SI ileum was further confirmed by *in situ* hybridization (ISH) (Figure S1b). Mice treated with BAA-835 showed increased crypt height and higher numbers of mucin-producing goblet cells in the SI and colon than seen in mice treated with PBS (Figure 1a and S2a). Owing to the increased presence of goblet cells in response to BAA-835 treatment, Mucin 2 (Muc2) protein and mRNA expression were examined in the SI. Muc2 protein and mRNA expression, along with mucus thickness, were significantly higher in the SI of BAA-835-treated mice than in PBS-treated control mice (Figures 1b and 1c). *Muc2* mRNA expression was also enhanced in colon of BAA-835-treated mice (Figure S2b). Additionally, administration of BAA-835 resulted in increased numbers of lysozyme-positive (Lyz^+^) Paneth cells and mRNA expression of *Lyz1* in the crypt of the SI (Figures 1d and 1e). Previously, Lgr5^+^Ki67^−^ cells located at crypt positions +4/+5 in the SI were found to differentiate into secretory lineage cells.^36^ In the current study, BAA-835-treated mice had significantly more Lgr5^+^Ki67^−^ cells at the +4/+5 crypt positions than PBS-treated mice (Figures 1f and 1g). As predicted, we observed an increase in the expression of the transcription factors, *Dll1, Math1*, and *Spdef1*, that regulate differentiation of secretory lineage cells^37-39^ in BAA-835-treated mice compared with controls (Figure 1h). These results suggest that *A. muciniphila* may promote differentiation of secretory lineage cells in the SI.

**(a)** PAS staining of the SI and quantification of goblet cell numbers and crypt heights. (**b**) The SIs of mice treated with BAA-835 were fixed by water-free methanol Carnoy’s fixative and stained with anti-Muc2 antibody to determine mucus thickness. (**c**) SI mRNA levels of *Muc2*. (**d**) Confocal image of Lysozyme^+^ Paneth cells and quantification. (**e**) SI mRNA levels of *Lyz1*. (**f**) Confocal image of SI stained by anti-Ki67 antibody. (**g**) Percentages of Lgr5 GFP^+^ cells either expressing Ki67 or not in SI crypt. (**h**) mRNA levels of *Dll1, Math1*, and *Spdef1*. Scale bars: 100 µm (**a**); 20 µm (**b**); 50 µm (**d**); 5 µm (**f**). Statistical analyses were done by two-tailed paired t-test. n = 3–5. *p < .05, **p < .01, ***p < .001. Data were combined from ≥3 independent experiments.

### Oral administration of A. muciniphila BAA-835 may accelerate ISC proliferation

**Figure 2.**
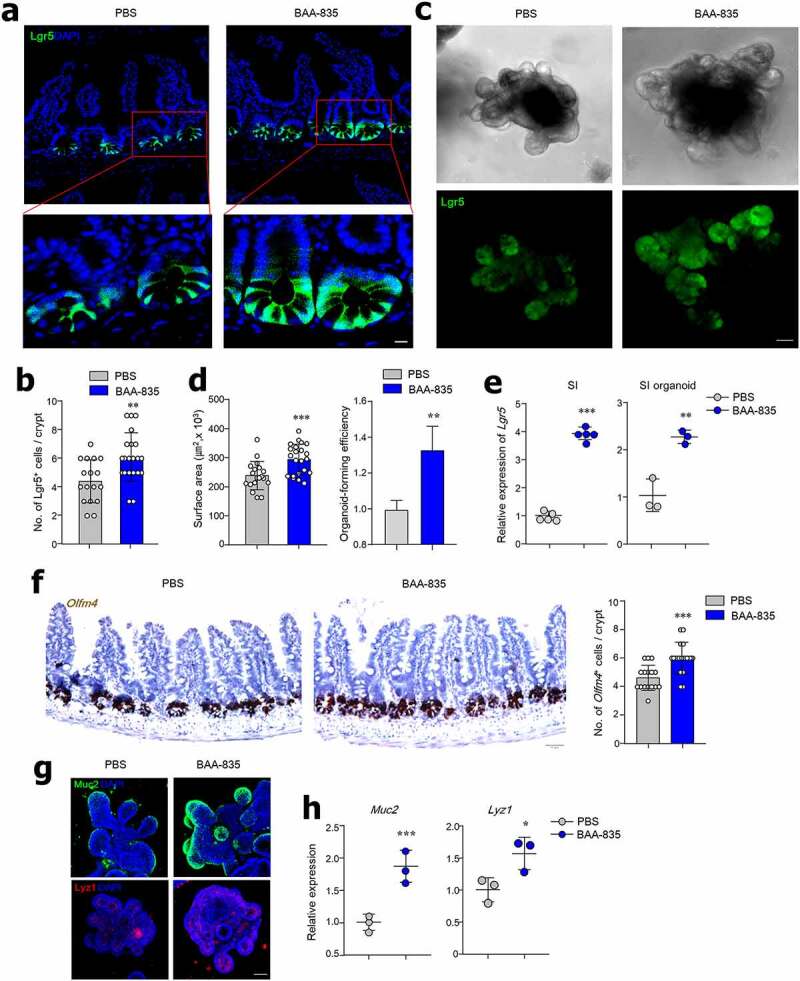
Mice treated with *A. muciniphila* BAA-835 showed enhanced Lgr5^+^ stem cell populations in SI and SI organoids

As secretory subtypes of IECs are derived from Lgr5^+^ ISCs, we next investigated whether *A. muciniphila* modulates the proliferation of Lgr5^+^ ISCs. Lgr5-GFP mice that were administered BAA-835 had more GFP-expressing Lgr5^+^ ISCs in the SI and colon crypt than PBS-treated mice (Figure 2a, 2b, and S2c). Organoids derived from the SI and colon crypt of BAA-835-treated mice were larger than those of PBS-treated mice (figure 2c, 2d, and S2d). Furthermore, *Lgr5* expression was upregulated in the SI, SI organoids, and colon of BAA-835-treated mice compared with controls (Figure 2e and S2e). RNA ISH analysis indicated increased numbers of *Olfm4*^+^ cells, another marker of ISCs, in the SI crypt of BAA-835-treated mice (Figure 2f). In addition, protein and mRNA levels of *Muc2* and *Lyz1* were upregulated in the SI organoids of BAA-835-treated mice compared with PBS-treated mice (Figures 2g and 2h). These results imply that *A. muciniphila* may play a critical role in accelerating the proliferation of ISCs.

Confocal images of Lgr5 GFP cells (**a**) and quantification of Lgr5 GFP cells (**b**) in SI crypts. Lgr5 GFP expression (**c**) and surface areas for determining organoid-forming efficiency (**d**) of SI organoids. (**e**) mRNA levels of *Lgr5* in SI tissues and organoids. Each individual point reflected pooled organoids from a single well. (**f**) RNA *in situ* hybridization for *Olfm4* and quantification of *Olfm4*^+^ cells in SI. (**g**) Confocal image of SI organoid stained by anti-Muc2 and anti-Lyz1 antibodies. (**h**) mRNA levels of *Muc2* and *Lyz1* in SI organoids. Each individual point reflected pooled organoids from a single well. Scale bars: 10 µm (**a**); 50 µm (**c**); 50 µm (**f**); 50 µm (Muc2); and 20 µm (Lyz) (**g**). Statistical analyses were carried out by two-tailed paired t-test, n = 3–5. *p < .05, **p < .01, ***p < .001. Data were combined from ≥2 independent experiments.

### Oral administration of A. muciniphila BAA-835 enhances ISC proliferation by Wnt signaling

**Figure 3.**
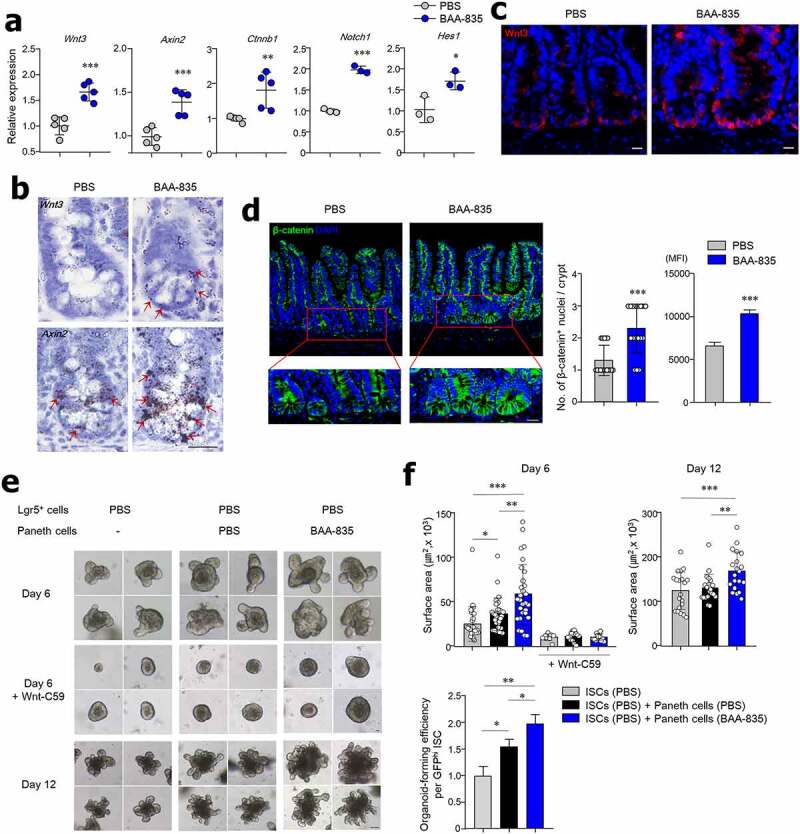
Administration of *A. muciniphila* BAA-835 activated Wnt/β-catenin pathway to enhance proliferation of ISCs

As Wnt and Notch signaling are involved in maintaining ISC stemness in the SI crypt, we next investigated whether *A. muciniphila* treatment regulates those pathways. Mice given BAA-835 orally had increased expression of *Wnt3, Axin2, Ctnnb1, Notch1*, and *Hes1* in their SI tissues (Figure 3a). Although both Wnt (*Wnt3, Axin2, Ctnnb1*) and Notch (*Notch1* and *Hes1*) signaling were activated by oral BAA-835, we focused on the Wnt-related signal because previous studies suggested the most important pathway for IEC development is Wnt signaling.^6,40^ The upregulated expression of *Wnt3* and *Axin2* in the SI crypt of BAA-835-treated mice was further confirmed by RNA ISH (Figure 3b). In addition, Wnt3 protein levels were higher in the SI crypt and SI organoids of BAA-835-treated mice than in PBS-treated control mice (Figure 3c and S1c). Of note, β-catenin protein levels were upregulated in the nuclei of the SI crypt of BAA-835-treated mice compared with PBS-treated mice (Figure 3d), therefore indicating increased translocation from the cytoplasm. As the Wnt/β-catenin pathway activates RAS-ERK signaling that in turn promotes stemness,^41^ we next examined ERK phosphorylation (pERK) in the SI crypt. As expected, oral administration ofh BAA-835 increased pERK expression in the SI crypt compared with controls (Figure S1d). To address whether *A. muciniphila* activates Wnt3 signaling in the SI, Lgr5-GFP^hi^ ISCs isolated from naive mice were co-cultured with Paneth cells isolated from BAA-835-treated or PBS-treated mice. Interestingly, SI organoids grew significantly more in co-cultures isolated from BAA-835-treated mice than in PBS-treated mice (Figures 3e and 3f). This effect was diminished when the porcupine inhibitor (Wnt-C59) was added to the co-cultures (Figures 3e and 3f). Together, these findings demonstrate that *A. muciniphila* promotes the secretion of Wnt3 from Paneth cells that support ISC proliferation in the SI.

(**a**) mRNA levels of *Wnt3, Axin2, Ctnnb1, Nochi1*, and *Hes1* in SI tissues. (**b**) *In situ* hybridization for *Wnt3* and *Axin2* in SI crypts. (**c**) Confocal images of Wnt3 in SI. (**d**) Representative confocal images of β-catenin in SI (left); numbers of nuclear translocation and mean fluorescence intensity (MFI) of β-catenin (right). (**e**) Co-culture with Lgr5 GFP ISCs and Paneth cells in the presence of Wnt-C59. (**f**) Surface area of the organoids was scored six and 12 days after plating for determining organoid-forming efficiency on Day 6. Scale bars: 10 µm (**b**); 20 µm (**c, d**); 20 µm on Day 6 and 50 µm on Day 12 (**e**). Statistical analyses were carried out by two-tailed paired t-test (**a, d**) and one-way ANOVA with post hoc Tukey’s test (**f**). n = 3–5. *p < .05, **p < .01, *** *P* < .001. Data were combined from ≥3 independent experiments.

***Oral administration of A. muciniphila BAA-835 promotes SCFA secretion and ISC-mediated epithelial development.***

**Figure 4.**
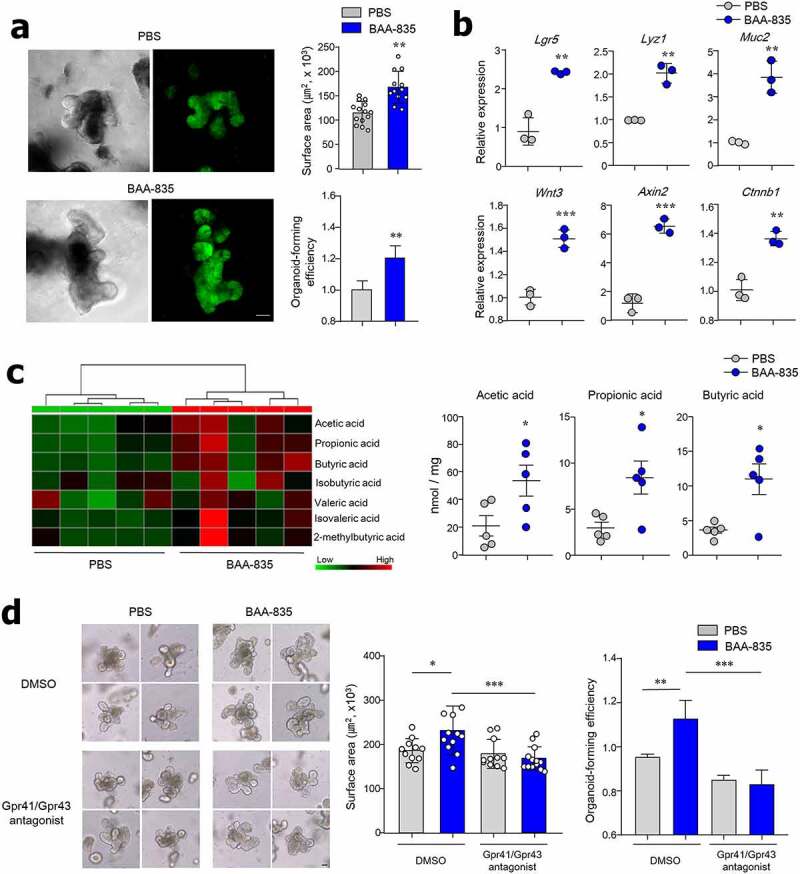
*A. muciniphila* BAA-835-derived metabolites promote SI organogenesis

Based on the fact that microbiota-derived metabolites are a key factor in gut homeostasis, we next investigated whether metabolites produced from *A. muciniphila* treatment affected ISC-mediated epithelial development. To address this question, the cecal contents from PBS- or BAA-835-treated mice were isolated and then applied to SI and colon organoids from naive B6 mice and humans. Mouse SI and colon organoids, and human colon organoids treated with BAA-835-treated mouse cecum were significantly larger than those treated with PBS-treated cecum (Figure 4a, S2f, and S2g). Furthermore, the mRNA levels of *Lgr5, Lyz1, Muc2*, and Wnt-related genes (*Wnt3, Axin2, Ctnnb1*) increased in the presence of cecal contents obtained from BAA-835-treated mice compared with PBS-treated mice (Figure 4b). To identify which metabolites are associated with *A. muciniphila*-mediated epithelial development, the levels of SCFAs were examined in cecal contents. As predicted, higher levels of SCFAs, including acetic, propionic, and butyric acids, were found in the cecal contents of BAA-835-treated mice than in PBS-treated mice (Figure 4c). Of these, acetic and propionic acids, but not butyric acid, were highly associated with increased SI organoid growth (Figure S3). Treatment with the Gpr41/43 antagonist reduced SI organoid growth and forming efficiency (Figure 4d), suggesting that BAA-835-derived SCFA metabolites play an important role in ISC-mediated epithelial development. In support of the indispensable role of BAA-835-derived secretory components, we found that oral administration of heat-inactivated BAA-835 completely eliminated the ability to activate genes related to ISC-mediated epithelial development (Figure S4).

(**a**) Expression of Lgr5 GFP and surface area of SI organoid in the presence of cecal contents. (**b**) mRNA levels of *Lgr5, Lyz1, Muc2, Wnt3, Axin2*, and *Ctnnb1* in SI organoids treated with cecal contents. Each individual point reflected pooled organoids from a single well. (**c**) Heat map of SCFA (left) and quantification of acetic, propionic, and butyric acids (right) derived from cecal contents. (**d**) Representative bright-field image, surface area, and forming efficiency of SI-derived organoid treated with cecal contents in the absence or presence of Gpr41/43 antagonists. Scale bars: 50 µm (**a**); 100 µm (**d**). Statistical analyses were performed by two-tailed paired t-test (**b, c**) and one-way ANOVA with post hoc Tukey’s test (**d**). n = 3–5. *p < .05, **p < .01, ***p < .001. Data were combined from ≥3 independent experiments.

***Oral administration of A. muciniphila BAA-835 alters gut microbiota composition and SCFA production.***

**Figure 5.**
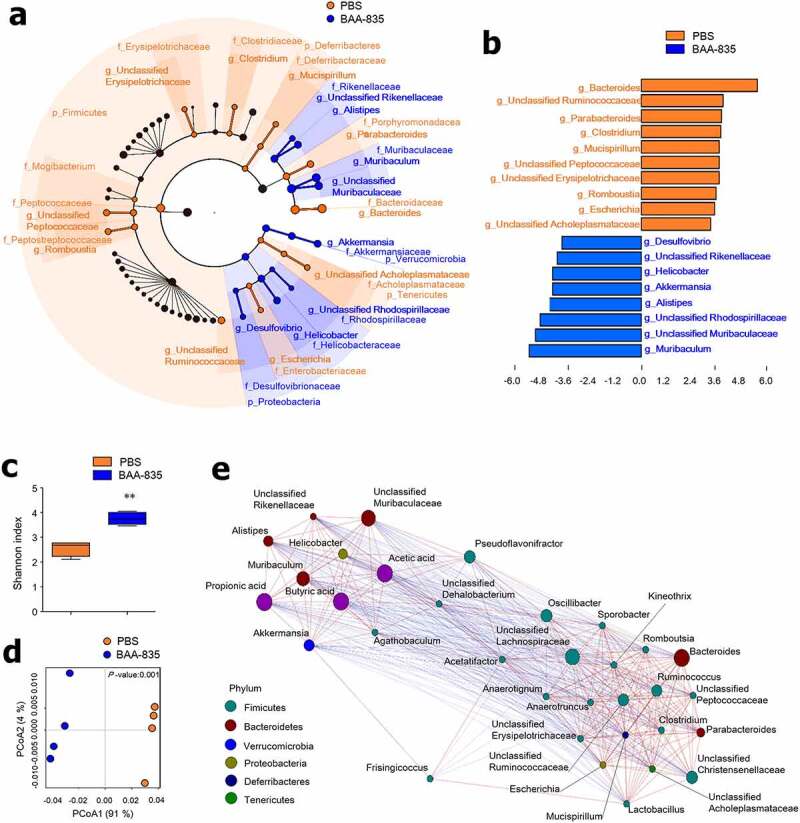
Administration of *A. muciniphila* BAA-835 results in change of gut microbiota composition and community structure

We next addressed whether oral administration of *A. muciniphila* altered the composition of gut microbiota and, as predicted, found that it did (Figure S5a). At the phylum level, the gut microbiota from BAA-835-treated mice exhibited an increased proportion of the phyla Bacteroidetes and Proteobacteria and decreased numbers of the phyla Firmicutes compared with PBS-treated mice (Figure S5b). Further, linear discriminant analysis (LDA) with LEfSe confirmed that several bacterial genera were prominently changed after BAA-835 treatment (Figures 5a and 5b). The genera *Muribaculum, Alistipes, Akkermansia, Helicobacter*, and *Desulfovibrio* showed an upper 2 LDA score after BAA-835 treatment compared with control mice (Figure 5b). Furthermore, the Shannon index was significantly increased in BAA-835-treated mice compared with PBS-treated mice, indicating alteration of the bacterial community structure (Figure 5c). Unifrac-based PCoA analysis demonstrated that the two groups were clustered separately (Figure 5d). Interestingly, there was a positive correlation between the BAA-835-induced population and the presence of SCFA metabolites (acetic, propionic, and butyric acids) (Figure 5e). In summary, *A. muciniphila* treatment might promote ISC-mediated epithelial development by altering the gut microbiota composition, which in turn activates SCFA secretion.

(**a**) Taxonomic cladogram from LEfSe. Dot size is proportional to taxon abundance. (**b**) LDA scores of differentially abundant taxa in fecal microbiome. LDA score > 2. (**c**) Shannon index in fecal microbiome. (**d**) PCoA of weighted UniFrac distances in fecal microbiome. (**e**) Correlation network between SCFAs and fecal microbiome. Dot color represents phylum level. Dot size is proportional to taxon abundance. Statistical analyses were performed by two-tailed paired t-test. n = 4. **p < .01. Data were combined from ≥2 independent experiments.

### Oral administration of A. muciniphila BAA-835 promotes epithelial differentiation in germ-free mice

To directly address a role of altered gut microbiota, we next adopted germ-free mice and orally administered BAA-835 twice per 2-week interval. Germ-free mice treated with BAA-835 showed increased crypt height and more mucin-producing goblet cells in the SI than did germ-free mice treated with PBS (Figure S6a). In addition, expression levels of *Lgr5, Lyz1, Muc2, Wnt3, Axin2*, and *Ctnnb1* in the SI tissues were significantly higher in BAA-835-fed germ-free mice than in PBS-fed germ-free mice (Figure S6b). These results suggest that oral *A. muciniphila* activates epithelial regeneration directly unlike other gut microbiota, which may be altered by *A. muciniphila.*

### Oral administration of A. muciniphila BAA-835 may repair radiation and chemotherapy gut damage

**Figure 6.**
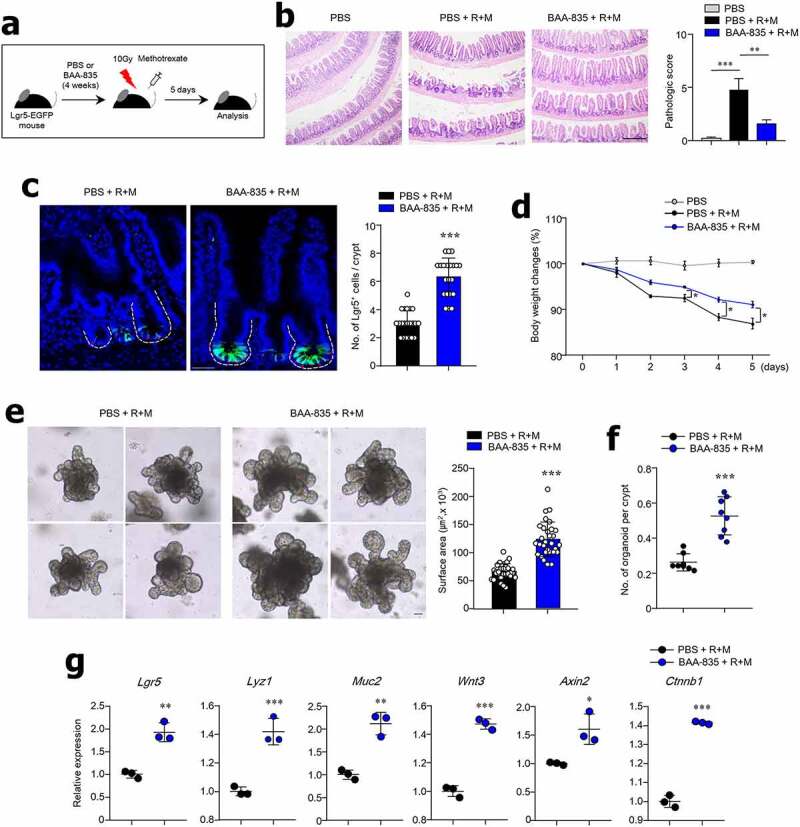
Administration of *A. muciniphila* BAA-835 prevents mouse gut injury

As *A. muciniphila* promotes ISC-mediated epithelial development, we next investigated whether *A. muciniphila* plays a role in preventing gut damage. Our previous study^22^ showed that radiation (R; 10 Gy) and methotrexate (M; MTX) cause severe damage to mouse SI tissues. In this study, we assessed PBS-treated mice (PBS+R+M) (Figures 6a and 6b) and mice treated with BAA-835 for 4 weeks prior to radiation and MTX treatment (BAA-835+R+M). The treated group had less severe damage (Figure 6b). In addition, more Lgr5^+^ ISCs were maintained in the SI crypt of BAA-835+R+M mice compared with the PBS+R+M mice (Figure 6c). As predicted, BAA-835 + R + M mice lost less weight than the PBS+R+M mice (Figure 6d). The organoid size and number derived from the SI of BAA-835+R+M mice were significantly increased in comparison with those of PBS+R+M-derived SI organoids, indicating that pre-treatment with BAA-835 reduced damage and may play a protective role in the gut (Figures 6e and 6f). Of note, BAA-835+R+M resulted in increased mRNA expression of *Lgr5, Lyz1, Muc2, Wnt3, Axin2*, and *Ctnnb1* in the SI tissues compared to PBS+R+M (Figure 6g). These results suggest that the symbiotic actions of *A. muciniphila* may promote gut repair following damage provoked by cancer therapy.

(**a**) Timeline of gut injury experiment. (**b**) Pathology and scores of SI by H&E staining. (**c**) Representative confocal image and number of Lgr5 GFP^+^ cells in SI following treatment with radiation (R) and methotrexate (M; MTX). (**d**) Body weight changes of mice after treatment with R and MTX. (**e**) Representative bright-field image and surface area of SI organoid mice treated with R and MTX. (**f**) Organoid-forming capacity of SI crypt obtained from mice after treatment with R and MTX. (**g**) mRNA levels of *Lgr5, Lyz1, Muc2, Wnt3, Axin2*, and *Ctnnb1* in SI tissues treated R and MTX. Scale bars: 100 µm (**b**); 50 µm (**c**); 50 µm (**e**). Statistical analyses were performed by two-tailed paired t-test (**d, f, g**) and one-way ANOVA with post hoc Tukey’s test (**b**). n = 3. *p < .05, **p < .01, ***p < .001. Data were combined from ≥2 independent experiments.

***A. muciniphila AK32 from healthy human feces is superior to BAA-835 for ISC-mediated epithelial development.***

**Figure 7.**
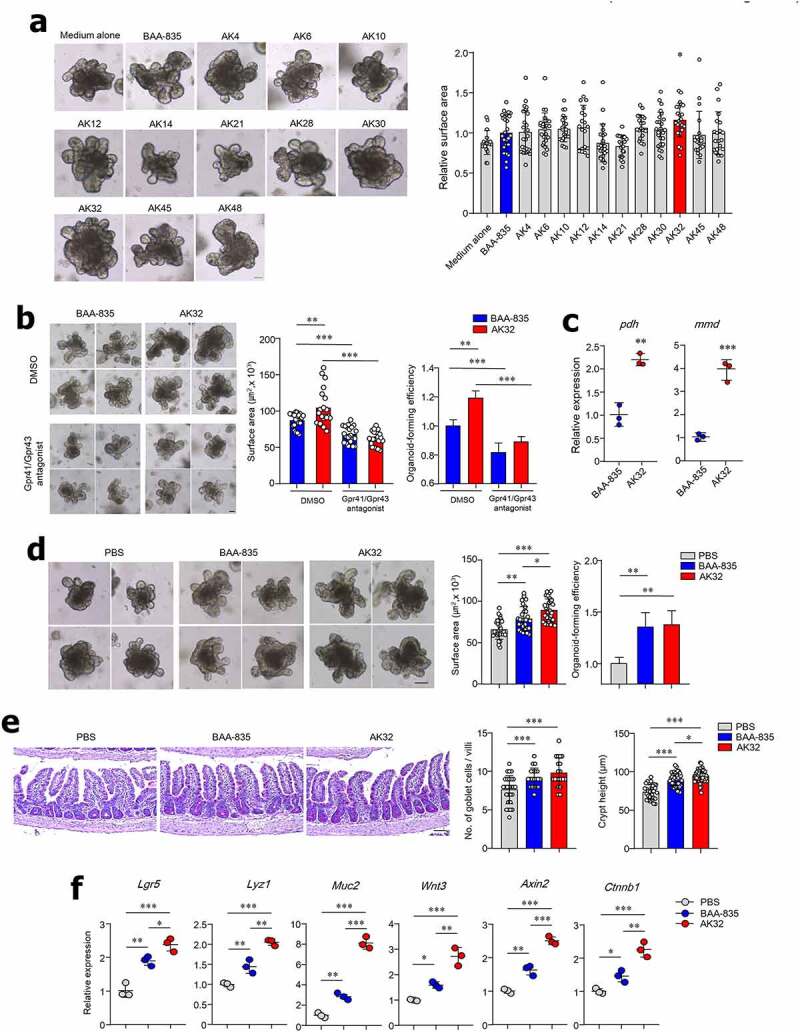
Isolation of *A. muciniphila* strains and validation of ISC stemness promoted by *A. muciniphila.*

We next investigated whether an *A. muciniphila* strain isolated from healthy human feces promotes ISC-mediated epithelial development compared with the BAA-835 type strain. By use of selective media and species-specific PCR analysis, we obtained 11 different *A. muciniphila* strains. To evaluate the effect of *A. muciniphila* on ISC-mediated epithelial development, SI-derived organoids were cultured with culture supernatant from one of the strains. Only treatment with the AK32 strain significantly increased organoid size (Figure 7a). To address whether increased ISC-mediated epithelial development by the AK32 strain is dependent on SCFAs, the Gpr41/43 antagonist was applied to the cultures. Treatment with the Gpr41/43 antagonist significantly reversed the AK32-mediated effect on SI-derived organoid size (Figure 7b). As anticipated, AK32 increased production of acetic acids and propionic acids compared with BAA-835 (Figure S7a). To examine how the AK32 enhanced SCFA secretion, the expression levels of two important enzymes, pyruvate dehydrogenase E1 component (Pdh) and Na^+^-translocating methylmalonyl-CoA/oxaloacetate decarboxylase (Mmd) were evaluated (Figure S7c). The mRNA expression levels of *pdh* and *mmd* from the AK32 strain were higher than those of the BAA-835 type strain (Figure 7c). Next, we used whole-genome sequencing to analyze the genetic characteristics of AK32, including the *pdh-* and *mmd*-coding genes. Figure S8 shows a complete genome map of the AK32-based Clusters of Orthologous Groups. The trimming information and the genomic characteristics (genome size, numbers of coding sequences, and ANIb) of the AK32 strain differed from those of BAA-835 (Tables S1 and S2). As the mRNA expression differences might be attributed to regulation of transcription by promoter sequences, we assessed the operons, including *pdh* and *mmd*. Interestingly, the strains AK32 and BAA-835 had the same promoter sequences and amino acid sequences of *pdh* but those of *mmd* differed (Figures S9 and S10). To further determine a novel strain of AK32, we compared the metabolic gallery (API 20A and API ZYM), MALDI-TOF spectrum profiles, growth capacity, and phylogenetic tree. Although AK32 did not differ from BAA-835 in terms of carbohydrate fermentation, AK32 expressed more α-, β-galactosidase than BAA-835 (Figures S11a and S11b). The matching spectrum with MALDI-TOF MS analysis showed some peaks that differed between AK32 and BAA-835 (Figures S11 c and S11d). The growth capacity of AK32 was identical to BAA-835 at an early stage while CFUs of the AK32 strain after 28 h were downregulated compared to those of BAA-835 (Figure S11e). The phylogenetic tree showed the AK32 strain to be closely related to BAA-835 (Figure S12). To further examine *in vivo* function, mice were treated with either the AK32 or BAA-835 strains for 4 weeks. Of note, the SI organoids from AK32-treated mice were significantly larger than those from mice treated with the BAA-835 strain (Figure 7d). Administration of AK32 increased SI crypt height and the number of mucin-producing goblet cells compared with the SI of mice treated with BAA-835 (Figure 7e). Administration of AK32 resulted in increased mRNA expression of *Lgr5, Lyz1, Muc2, Wnt3, Axin2*, and *Ctnnb1* in the SI compared with mice treated with BAA-835 (Figure 7f). Furthermore, higher levels of acetic and propionic acids were detected in the cecal contents of AK32-treated mice than in mice treated with BAA-835 (Figure S7b). Thus, we concluded that the newly identified AK32 strain was superior to the BAA-835 type strain in terms of ISC-mediated epithelial development.

(**a, b**) Representative bright-field image and surface area of SI organoids. (**a**) Treated with culture supernatant from BAA-835 or one of 11 *A. muciniphila* isolates. (**b**) Treated with bacterial culture supernatant in the absence or presence of Gpr41/43 antagonists. (**c**) mRNA expression levels of pyruvate dehydrogenase E1 component α subunit (*pdh*) and Na^+^ transporting methylmalonyl-CoA/oxaloacetate decarboxylase β subunit (*mmd*) in BAA-835 or AK32 strains. (**d**) Representative bright-field image, surface area, and forming efficiency of SI-derived organoids from mice treated with PBS or the BAA-835 or AK32 strains. (**e**) Quantification of goblet cells and crypt height in mouse SI. (**f**) mRNA levels of *Lgr5, Lyz1, Muc2, Wnt3, Axin2*, and *Ctnnb1* in SI tissues. Scale bars: 50 µm (**a, b**); 100 µm (**d**); 50 µm (**e**). Statistical analyses were performed by two-tailed paired t-test (**c**) and one-way ANOVA with post hoc Tukey’s test (**a, b, d, e, f**). n = 3–5. *p < .05, **p < .01, ***p < .001. Data were combined from ≥2 independent experiments.

## Discussion

In this study, *A. muciniphila* was found to play a crucial role in ISC-mediated epithelial development by activation of the Wnt signaling pathway and repair of the damaged gut. Treatment with *A. muciniphila* upregulated the expression of genes involved in the Wnt signaling pathway and increased production of SCFA metabolites, such as acetic and propionic acids, which in turn maintain the stemness of ISCs. A novel *A. muciniphila* strain was isolated from healthy human stools that promoted the expression of genes involved in acetic acid and propionic acid production, and therefore may have improved functionality for maintaining gut homeostasis.

The Lgr5^+^Ki67^−^ cells located at crypt positions +4/+5 of the SI are destined to differentiate into secretory lineage cells.^36^ Administration of *A. muciniphila* resulted in an increased density of Lgr5^+^Ki67^−^ cells in the SI crypt, which led to an increased number of secretory lineage cells, such as goblet and Paneth cells. The most important pathway for IEC development is Wnt signaling.^6,40^ In support of this theory, inhibition of the Wnt pathway was shown to reduce the number of Math1^+^ precursor cells, resulting in a depletion of secretory lineage cells.^7^ Paneth cells play a critical role in maintaining the ISC niche and the production of Wnt3.^5^ In this study, the co-culture of ISCs and Paneth cells demonstrated that Paneth cells from *A. muciniphila*-treated mice directly promoted ISC proliferation in a Wnt3-dependent manner. Thus, we speculate that metabolites produced by *A. muciniphila* stimulate Paneth cells to secrete Wnt3, which then promotes the proliferation of Lgr5^+^Ki67^−^ cells at SI crypt positions +4/+5.

A previous study reported that *A. muciniphila* uses mucin as an energy source by converting it into acetic and propionic acids.^42^ Paradoxically, others have found that *A. muciniphila* promotes the generation of mucin-secreting goblet cells that were depleted by a high-fat diet.^27,33,43^ In this study, our aim was to investigate how *A. muciniphila* activates mucin secretion. We propose that acetic and propionic acids produced by *A. muciniphila* may be key factors for supporting the maturation of mucin-secreting goblet cells.

Another study reported that administration of *A. muciniphila* did not reconstitute the gut microbiome.^32^ In contrast, we found that *A. muciniphila* altered the gut microbiota composition and structure, which may affect the pattern of metabolite secretion. Germ-free mice supplemented with *A. muciniphila* had an exacerbated infection by *Salmonella typhimurium*, suggesting that an over-abundance of *A. muciniphila* diminished microbiome diversity, leading to a deleterious modification of the gut environment.^44^ Furthermore, an accumulation of antimicrobial peptide produced by Paneth cells may contribute to a change in gut microbiota.^45^ We therefore hypothesized that treatment with *A. muciniphila* may alter the bacterial composition and SCFA production in the gut; for example, by altering the abundance of the phyla Bacteroidetes, which produces acetic and propionic acids,^46^ or *Alistipes* and *Rikenellaceae*, which produce SCFAs.^47,48^ Unexpectedly, however, oral administration of *A. muciniphila* in germ-free mice resulted in enhancement of ISC-mediated epithelial development (Figure S6). Therefore, we conclude there might be two pathways through which *A. muciniphila* increases SCFA production, directly via mucin degradation and indirectly by altering microbiome composition.

Individual bacterial strains, even the same species, have strain-specific abilities. As lactic acid bacterial strains of the same species show different enzymatic activities,^49^ we wondered whether different *A. muciniphila* strains might have varying effects on ISC-mediated epithelial development. We found that the newly identified *A. muciniphila* AK32 strain activated the expression of genes involved in SCFA production and increased the secretion of acetic and propionic acids. As expected, promoter sequences of *mmd* in AK32 differed by type strain, but those of *pdh* were highly conserved. The detailed mechanism by which expression occurs is yet to be determined. We did identify a strain-specific alteration of the Mmd amino acid sequence, which may influence enzyme activity by altering propionic acid levels. Thus, we conclude that strain AK32 has a characteristic genome, unlike the *A. muciniphila* type strain, leading to increased SCFA production, especially acetic and propionic acids.

Treatment with *A. muciniphila* had a greater effect on the production of propionic acid than on acetic acid. Previously, it was suggested that *A. muciniphila* and propionic acid regulate the expression of genes associated with the host lipid metabolism and activate the epigenome.^50^ Accumulating evidence suggests that propionic acid may modulate host physiology in several ways. For example, propionic acid stimulates the release of peptide YY and glucagon-like peptide-1 in human colonic cells, and thereby reduces energy intake and weight gain.^51^ Intriguingly, propionic acid stimulates Muc2 production by IECs by regulating the expression of the prostaglandins.^52^ A recent study proposed that supplementation of propionic acid improves the Treg/Th17 imbalance in multiple sclerosis patients.^53^ Taken together with our results, we conclude that propionic acid may play an important role in IEC homeostasis and the overall gut, and therefore may modulate host physiology.

Several studies have reported an interaction between gut metabolites and IEC development.^22,54^ Our prior study revealed that microbiota-derived lactate promotes IEC development.^22^ In contrast, no significant changes in lactate were found in mice treated with *A. muciniphila*, suggesting that lactate is not a crucial metabolite involved in *A. muciniphila*-mediated IEC development (Figure S13a). A previous study reported that fatty acids, including palmitic acid, the main metabolite produced by gut microbiota, enhanced ISC proliferation.^54^ Furthermore, our recent study demonstrated that dietary cellulose prevented gut inflammation by increasing the *A. muciniphila* population and modulating production of lipid metabolites.^55^ Taken together, *A. muciniphila* treatment may elevate the production of lipid metabolites, including myristic and palmitic acid, which influence IEC development (Figure S13b). Further investigation is warranted to rule out this possibility.

A previous study showed intestinal microbiota epigenetically modulated intestinal homeostasis.^56^ In colorectal cancer, colon dysbiosis caused methylation of host genes.^57^ Another study reported commensal bacteria-derived SCFAs regulated differentiation of colonic Treg by affecting histone acetylation.^17^ Although we focused on alteration of genetic patterns of IECs by *A. muciniphila* in this study, we speculated that epigenetic pathways might be involved. Further study is required to clarify epigenetic-related issues.

Our study findings indicate that *A. muciniphila* likely plays a crucial role in IEC development and may be a potential clinical therapeutic agent for preventing gut damage. To our knowledge, this is the first study to show the direct effects of *A. muciniphila* on ISC-mediated epithelium development. A new *A. muciniphila* strain was identified from healthy human stools with improved homeostatic functionality, such as increased production of acetic and propionic acids. Furthermore, this study may serve as a valuable basis for identifying and evaluating human microorganisms with therapeutic potential.

## Materials & methods

### Ethics statement

All animal experiments were approved by the Institutional Animal Care and Use Committee of Asan Medical Center (Approval No. 2019–12-251). Fecal samples and colon tissues were obtained from the human dock center of the Asan Medical Center under Institutional Review Board (Approval No. A20201614). All experiments were performed under anesthesia with a mixture of ketamine (100 mg/kg) and xylazine (20 mg/kg).

### Mice

The mice in this study were 8- to- 10-week-old females that were fed sterile food and water *ad libitum*. C57BL/6 mice were purchased from OrientBio (Seong-Nam, South Korea) and Lgr5-EGFP-IRES-CreERT2 (Lgr5-GFP) mice were purchased from The Jackson Laboratory (Bar Harbor, ME). Mice were housed in the animal facility of the Asan Medical Center (Seoul, South Korea) and maintained under specific pathogen-free conditions. Germ-free mice were maintained in the animal facility at POSTECH (Pohang, South Korea). All experiments were performed in accordance with relevant ethical guidelines and regulations.

### Isolation of A. muciniphila strains from human stool

A total of 32 fecal samples were obtained from the human dock center of the Asan Medical Center. The samples were collected from fresh residual samples after fecal occult blood and parasitic examination on the same date. Fecal samples were suspended in PBS and then seeded onto brain heart infusion agar without dextrose (Kisan-Bio, Seoul, South Korea) supplemented with 0.4% mucin (BHI-M). The fecal cultures were maintained at 37°C under anaerobic conditions generated using a GasPak 100 system (BD Bioscience). Approximately 50 colonies were selected from BHI-M plates and tested with PCR for species-specific sequences with the primer set, 5′-CAGCACGTGAAGGTGGGGAC-3′ and 5′-CCTTGCGGTTGGCTTCAGAT-3′. *A. muciniphila* was isolated from 11 samples. One strain was established per sample by sub-culturing for future experimentation.

### Oral administration of A. muciniphila

*A. muciniphila* (ATCC BAA-835 ^T^) and newly isolated AK32 (KCTC 14172BP) strains were cultured in brain heart infusion media (BD Bioscience) supplemented with 0.4% mucin (Sigma) and maintained in an anaerobic incubator using the GasPak 100 system (BD Bioscience) at 37°C. Cultures were centrifuged, the culture pellet was suspended in anaerobic PBS, and the culture pellet was orally administered to mice (8 × 10^8^ CFU per dose) every day for 4 weeks by a Zonde needle. Heat-inactivated BAA-835 was prepared by pasteurization for 30 min at 70°C^29^ and administered to mice orally (8 × 10^8^ CFU per dose).

### Treatment with irradiation and methotrexate

Mice were injected intraperitoneally with methotrexate (MTX; 120 mg/kg, Sigma) followed by administration of 10 Gy of total body irradiation (cesium source irradiator; Precision X-Ray, North Branford, CT).

### Cell isolation

Mouse SIs were opened longitudinally and washed with PBS. To dissociate the crypts, tissues were incubated at 4°C in 1 mM EDTA in PBS for 30 min, washed in PBS, and then transferred into 5 mM EDTA in PBS for an additional 1 h of incubation at 4°C. Samples were then suspended in PBS and filtered by a 70-μm cell strainer (BD Falcon). To purify ISCs and Paneth cells from Lgr5-GFP mice, crypt-cell suspensions were dissociated using TrypLE Express (Thermo Fisher Scientific) for 10 min at 37°C. The dissociated cells were stained with the Live/Dead Cell Stain kit (Thermo Fisher Scientific) and anti-CD24 monoclonal antibody (Thermo Fisher Scientific). Cell sorting was performed using a FACS Aria III cell sorter. ISCs were sorted as Lgr5-GFP^hi^ and Paneth cells were sorted as Lgr5-GFP^−^CD24^hi^, respectively.

### Organoid culture

For construction of organoids, 200–500 crypts per well were suspended in Matrigel (Corning) as described.^58^ Complete ENR medium (all components from Thermo Fisher Scientific unless noted) were comprised of advanced DMEM/F12 (Gibco), antibiotic-antimycotic (×100), 1 mM N-acetyl cysteine (Sigma-Aldrich), B27 supplement, N2 supplement, EGF, Noggin (R&D Systems), R-spondin-1-conditioned medium, and Y-27632 (Sigma). Y-27632 was added to the ENR medium for the first 48–72 h of culture only and then removed during the medium change. The ENR medium was replaced every 2 to 3 days. Colon organoids were cultured in the ENR medium supplemented with Wnt3 conditioned media (WENR). Human colon organoids were cultured in WENR medium supplemented with gastrin, nicotinamide, A83-01, and SB202190 (all from Sigma) as described.^59^ Isolated ISCs and Paneth cells were co-cultured in ENR medium supplemented with Jagged-1 (1 µM; Anaspec). Wnt-C59 (50 µM; Abcam) was used as a porcupine (PORCN) inhibitor. The surface areas of SI and colon organoids were measured microscopically by taking several random non-overlapping photos of organoids in a well using an inverted microscope (Carl Zeiss). Each photo was analyzed using ImageJ software (NIH) and the Zen image program (Carl Zeiss). Organoid perimeters for area measurements were defined manually using automated ImageJ software.

### Histology

Ileum tissues were removed, opened longitudinally, and formed into Swiss rolls. The tissue was then fixed in 4% paraformaldehyde (PFA) and embedded in paraffin. Tissue sections were stained with hematoxylin-eosin (H&E) or periodic acid-Schiff (PAS). Quantification of the crypt height and goblet cell number was carried out by blinded operators. Pathology scoring was conducted in a blinded fashion using a scoring system. In brief, two parameters were measured: extent of injury (0, none; 1, basal one-third damaged; 2, basal two-thirds damaged; 3, only surface epithelium intact; 4, entire crypt and epithelium lost) and crypt damage (0, none; 1, basal one-third damaged; 2, basal two-thirds damaged; 3, only surface epithelium intact; 4, entire crypt and epithelium lost). The sum of the two parameter values was multiplied by a factor that reflected the percentage of tissue involvement (1, 0%~25%; 2, 26%~50%; 3, 51%~75%; 4, 76%~100%).

### Immunofluorescence staining

Ileum tissues were fixed with 4% PFA and dehydrated with 15%, and then 30% sucrose in PBS. Dehydrated tissues were formed into a Swiss roll, frozen, and sliced. For staining, collected organoids were permeabilized in PBS containing 0.1% Tween 20 and blocked with 0.5% BSA in PBS for 1 h. For Muc2 staining, ileum tissues containing feces were fixed in Carnoy’s solution and embedded in paraffin. The primary antibodies used were rabbit anti-Muc2 (Abcam), rat anti-Ki67 (Biolegend), rabbit anti-lysozyme (Abcam), goat anti-Wnt3 (Abcam), mouse anti-β-catenin (BD Bioscience), and mouse anti-phospho-ERK1/2 (Thermo Fisher Scientific). Secondary antibodies were Alexa Fluor goat 594 anti-rat IgG (Biolegend), Alexa Fluor 488 goat anti-mouse IgG (Abcam), Alexa Fluor 546 donkey anti-goat IgG (Thermo Fisher Scientific), and Alexa Fluor 488 goat anti-rabbit IgG (Thermo Fisher Scientific).

### Organoid treatment with cecal contents and bacterial culture supernatants

Cecal contents (100 mg) were diluted in 1 mL of serum-free DMEM/F12 (Gibco) medium and vortexed for 1 h. The contents were centrifuged at 4,000 rpm for 10 min and supernatants were passed through a 0.22-·m syringe filter (Pall Corp.) before cultivation. Bacterial culture supernatants were prepared by centrifuging at 12,000 rpm for 10 min and passing the supernatants through a 0.22-·m syringe filter (Pall Corp.). To address the effectiveness of cecal contents or bacteria culture supernatants in promoting organogenesis, we used ENR or WENR media supplemented with cecal content supernatant (0.01%) or bacterial culture supernatant (4%) diluted in advanced DMEM/F12. For inhibition of Gpr41/43, we used Gpr43 antagonist (GLPG0974; 0.1 µM, Tocris) and Gpr41 antagonist (β-hydroxybutyrate, 3 mM; Sigma).

### Microbiome data analysis pipeline

Total DNA was extracted from feces and SI contents using QIAamp DNA stool mini kits (Qiagen) in accordance with the manufacturer’s instructions. For bacterial PCR amplification, primers targeting 341 F and 805 R were used. The amplified product was purified and sequenced by Chunlab (Seoul, South Korea) with an Illumina Miseq Sequencing system (Illumina). The processing of raw reads started with a quality check and filtering of low-quality (<Q25) reads by Trimmomatic software (ver. 032). After a quality control pass, paired-end sequence data were merged together using VSEARCH version 2.13.4 with default parameters. Nonspecific amplicons that did not encode 16S rRNA were detected by nhmmer in the HMMER software package, version 3.2.1. We used the EzBioCloud 16S rRNA database for taxonomic assignment by precise pairwise alignment.^60^ After chimeric filtering, reads that were not identified to the species level (with <97% similarity) in the EzBioCloud database were compiled. Operational taxonomic units with single reads (singletons) were omitted from further analysis. The alpha diversity (Shannon index) and beta diversity for the sample difference were estimated. A taxonomic cladogram was generated using LEfSe with a threshold of 2 on the logarithmic LDA score.^61^ A relationship based on a Pearson correlation between gut microbiota and SCFAs was visualized using Calypso software.^62^

### Whole-genome sequencing

The integrity of gDNA was tested by running an agarose gel electrophoresis. gDNA was quantified using the Quant-IT PicoGreen protocol (Invitrogen). The sequencing libraries were then prepared according to the manufacturer’s instructions (20 kb template preparation and the BluePippin^TM^ Size-Selection System) using the PacBio DNA template Prep Kit 1.0. The libraries were quantified using Quant-IT PicoGreen and qualified using a high-sensitivity DNA chip (Agilent Technologies). Subsequently, the libraries were sequenced using PacBio P6C4 chemistry in 8-well-SMART Cell v3 in PacBio RSII. The genome of the AK32 strain was constructed *de novo* using PacBio sequencing data. Sequencing analysis was performed by Chunlab. PacBio sequencing data were assembled with PacBio SMRT analysis 2.3.0 using the HGAP2 protocol (Pacific Biosciences). Resulting contigs from the PacBio sequencing data were circularized using Circlator 1.4.0 (Sanger Institute). The gene-finding and functional annotation pipeline of the whole-genome assembly was used from the EzbioCloud genome database. Protein-coding sequences were predicted by Prodigal 2.6.2.^63^ Genes coding for tRNA were searched using tRNAscan-SE 1.3.1.^64^ We searched the rRNA and other non-coding RNAs in the Rfam 12.0 database.^65^ Comparative whole-genome analysis was studied by average nucleotide identity base BLAST (ANIb). The ANIb value was calculated by ANI calculator from the Kostas lab (http://enve-omics.ce.gatech.edu/ani). Operons including *pdh* and *mmd* were predicted by the Microbes Online Operon Predictions tools.^66^ We estimated the −10 and −35 regions in promoter sequences of these genes through the phiSITE database.^67^

***Identification of A. muciniphila by carbohydrate fermentation pattern, enzymatic activity, and protein***

We determined the carbohydrate fermentation pattern and enzymatic activity, respectively, of API 20A and API ZYM (BioMérieux, France) per the manufacturer’s instructions. . MALDI-TOF MS analysis using the Microflex LT/SH (Bruker, France) was used for protein-based identification. Bacteria were prepared for MALDI-TOF MS analysis by using ethanol/formic acid extraction methods according to the manufacturer’s manual. Mass spectra were analyzed by MALDI Biotyper software package. Identification log scores were interpreted as recommended by the manufacturer. Log scoring was as follows: ≥2 and <3, high-confidence identification; ≥1.70 and <2, low-confidence identification; and ≥0 and <1.70, no organism identification possible.

### Real-time PCR

Total RNA from the SI, LI, and SI-derived organoids was extracted using the RNeasy mini kit (Qiagen) and cDNA was synthesized using Superscript ll reverse transcriptase and oligo dT primer (Thermo Fisher Scientific). Total RNA of *A. muciniphila* was extracted using Trizol (Thermo Fisher Scientific). The ReverTra Ace qPCR RT master mix with gDNA remover (Toyobo) was used to synthesize cDNA from bacterial RNA. cDNA was used as the template for real-time PCR performed using SYBR green chemistry (Thermo Fisher Scientific) on a Real-time PCR system (Applied Biosystems). The real-time PCR primers used in this study were as follows: *Muc2*, 5′-CCTTAGCCAAGGGCTCGGAA-3′ and 5′-GGCCCGAGAGTAGACCTTGG-3′; *Lyz1*, 5′-ATGGCGAACACAATGTCAAA-3′ and 5′-GCCCTGTTTCTGCTGAAGTC-3′; *Dll1*, 5′-CAGGACCTTCTTTCGCGTAT-3′ and 5′-AAGGGGAATCGGATGGGGTT-3′; *Math1*, 5′-GCCTTGCCGGACTCGCTTCTC-3′ and 5′-TCTGTGCCATCATCGCTGTTAGGG-3′; *Spdef1*, 5′-CCGGTTGCCTGCTACTGTTC-3′ and 5′-GCCCATTGCTCCTGATGCT-3′; *Wnt3*, 5′-CTTCTAATGGAGCCCCACCT-3′ and 5′-GAGGCCAGAGATGTGTACTGC-3′; *Axin2*, 5′-AACCTATGCCCGTTTCCTCT-3′ and 5′-GAGTGTAAAGACTTGGTCCA-3′; *Ctnnb1*, 5′-ATGGAGCCGGACAGAAAAGC-3′ and 5′-TGGGAGGTGTCAACATCTTCTT-3′; *Lgr5*, 5′-CCTGTCCAGGCTTTCAGAAG-3′ and 5′-CTGTGGAGTCCATCAAAGCA-3′; *Notch1*, 5′-GCTGCCTCTTTGATGGCTTCGA-3′and 5′-CACATTCGGCACTGTTACAGCC-3′; *Hes1*, 5′-CCAGCCAGTGTCAACACGA-3′ and 5′-AATGCCGGGAGCTACTTTCT-3′; *β-actin*, 5′-TGGAATCCTGTGGCATCCATGAAAC-3′ and 5′-TAAAACGCAGCTCAGTAACAGTCCG-3′; *pdh*, 5′-AACCGATTATTGAAGCGGCA-3′and 5′-ATATTGGCGGCTTCGTGAAA-3′; *mmd*, 5′-GACCAAGAAGGAACGCCTCA-3′ and 5′-GTTCCGTCACCTTGCATTCG-3′; Universal 16S rDNA, 5′-ACTCCTACGGGAGGCAGCAG-3′ and 5′-ATTACCGCGGCTGCTGG-3′; and *A. muciniphila* 16S rDNA, 5′-CAGCACGTGAAGGTGGGGAC-3′ and 5′-CCTTGCGGTTGGCTTCAGAT-3′.

### Quantitative measurement of SCFAs

All reagents and solvents for metabolite analysis were purchased from Sigma. Freeze-dried cecal contents (10 mg) were homogenized vigorously with 400 µL of internal standard solution [1 mM propionic acid (C_3_)-d_6_ and 100 µM butyric acid (C_4_)-d_7_] in water. For analysis of bacterial culture supernatant, we mixed 100 µL of culture supernatant with 200 µL of internal standard solution. After centrifuging, the supernatant was filtered out. AABD-SH (20 µL of 20 mM), TPP (20 µL of 20 mM), and DPDS (20 µL) in dichloromethane were added to the filtrate. The solution was incubated for 10 min at RT with vortexing and dried under vacuum. The sample was reconstituted with 80 μL of methanol prior to LC-MS/MS analysis. The LC-MS/MS system was equipped with a 1290 HPLC (Agilent Technologies, Denmark), Qtrap 5500 (ABSciex), and a reverse-phase column (Pursuit 5 C18 150 × 2.0 mm; Agilent Technologies). The extracted ion chromatogram (EIC) corresponding to a specific transition for each metabolite was used for quantitation. The area under the curve of each EIC was normalized to the EIC of the internal standard. The peak area ratio of each metabolite was normalized to the internal standard using serum volume or tissue weight in a sample, and then used for relative comparison.

### Statistics

Statistical analyses were performed by using Prism software (GraphPad, La Jolla) with a two-tailed t-test and one-way analysis of variance (ANOVA) followed by Tukey’s post hoc test. Data are presented as mean ± SEM. The values *p* < .05, *p* < .01, and *p* < .001 were considered statistically significant.

## Supplementary Material

Supplemental Material

## Data Availability

All microbiome data and whole-genome sequences are publicly available in the NCBI BioProject ID PRJNA625127, PRJNA632722, and PRJNA642315.
