# Supplementary material for: Mucin degrader Akkermansia muciniphila accelerates intestinal stem cell-mediated epithelial development
Source: Gut Microbes. 2021 Mar 8;13(1):1892441. doi: 10.1080/19490976.2021.1892441 (PMC7946046; doi:10.1080/19490976.2021.1892441)
Supplement: Supplemental Material [file KGMI_A_1892441_SM4275.pdf]

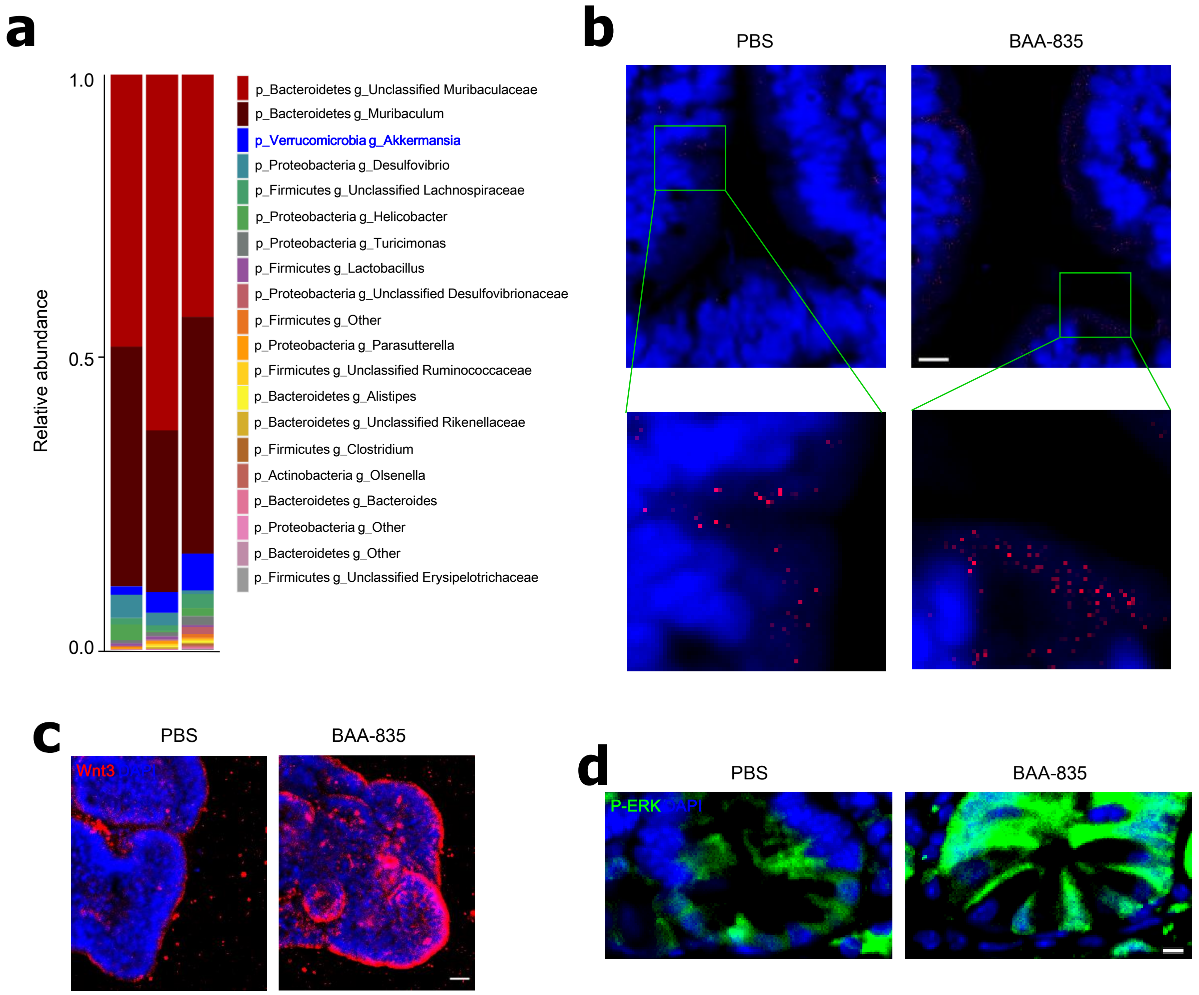

Figure S1. Bacteria colonization in mouse SI after administration of *A. muciniphila* BAA-835. (**a**) Microbiota composition of SI contents from naïve B6 mice at the genus level. (**b**) Fluorescence *in situ* hybridization of *A. muciniphila* in SI. Ileum tissues of BAA-835-treated mice were fixed in Carnoy's solution (methanol : chloroform : acetic acid = 6 : 3 : 1) and embedded in paraffin. Tissue sections were hybridized with the Cy-3-labeled fluorescence probe specific for *A. muciniphila* 16s rDNA (5′-CCTTGCGGTTGGCTTCAGAT-3′) in hybridization solution (20 mM Tris, pH 7.4, 0.9 M NaCl, 0.1% sodium dodecyl sulfate). (**c**, **d**) Representative confocal image of SI-derived organoid stained by anti-Wnt3 antibody and SI tissue stained by anti-P-ERK antibody. Scale bars: 10 μm (**b**); 50 μm (**c**); 20 μm (**d**). n=3.

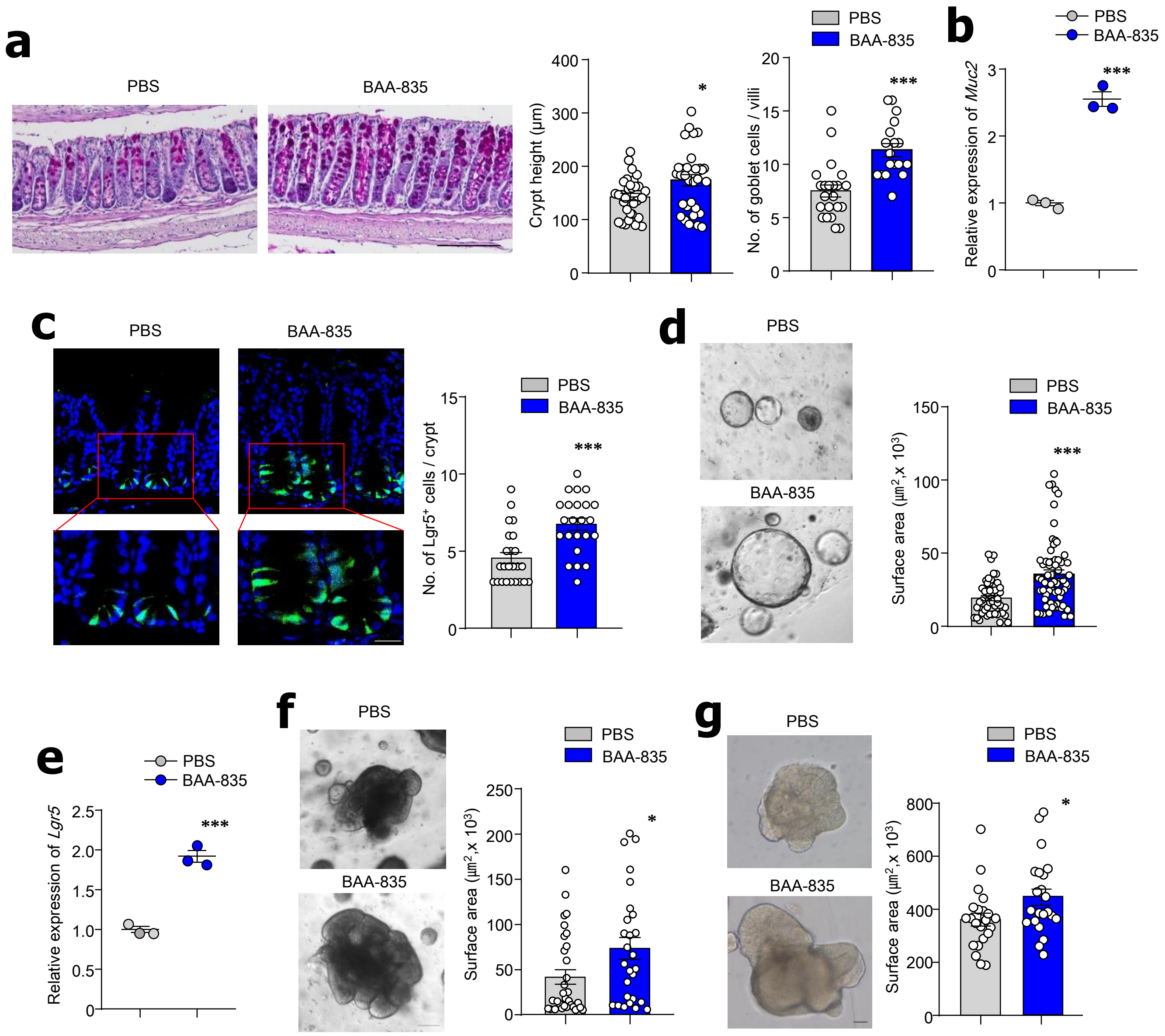

Figure S2. Mice treated with *A. muciniphila* BAA-835 had enhanced development in colon. (**a**) PAS staining of the colon and quantification of goblet cell numbers and crypt heights. (**b**) Colon mRNA levels of *Muc2*. (**c**) Confocal images of Lgr5 GFP cells and quantification of Lgr5 GFP cells in colon crypts. (**d**) Representative bright-field image and surface area of colon-derived organoids. (**e**) Colon mRNA levels of *Lgr5*. (**f**) Surface area of mouse colon organoid in the presence of cecal contents. (**g**) Surface area of human colon organoid in the presence of cecal contents. Scale bars: 200 μm (**a**); 20 μm (**c**); 100 μm (**e**); 100 μm (**f**); 100 μm (**g**). Statistical analyses were performed by two-tailed paired t-test (**b**). n=3. *$p < 0.05$, **$p < 0.01$, ***$p < 0.001$.

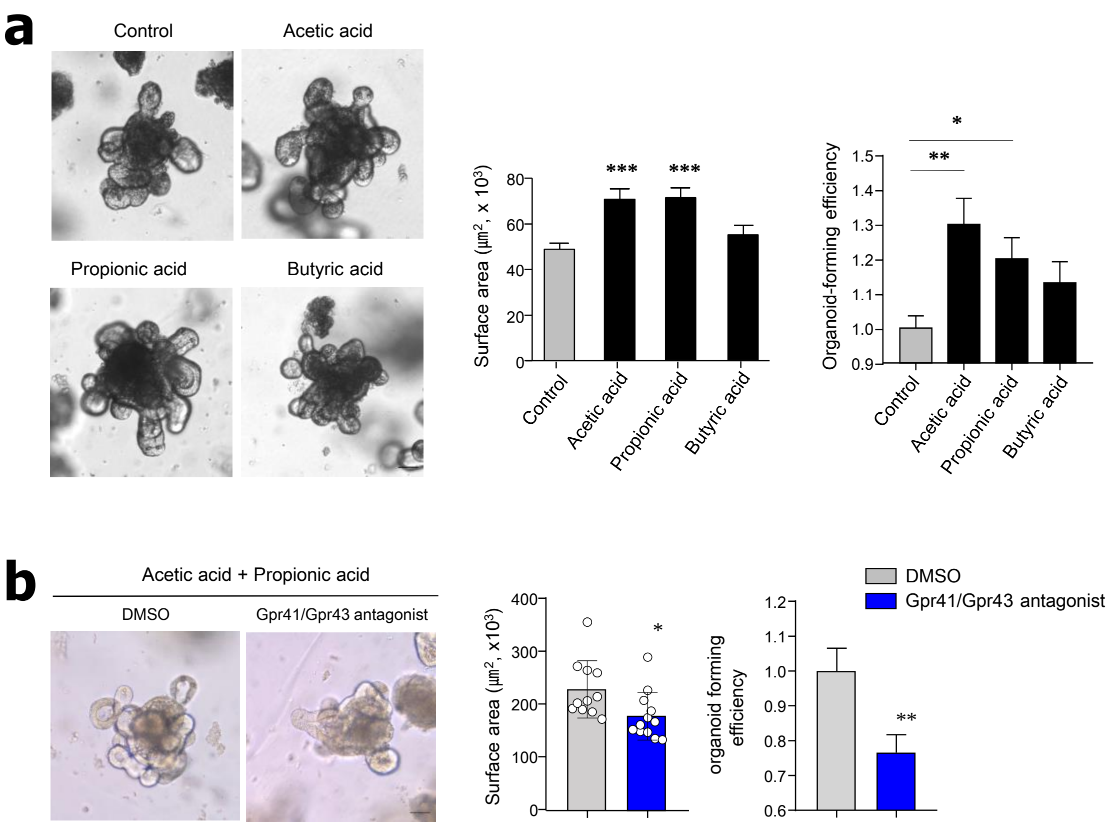

Figure S3. SI organoid growth measured after SCFA treatment. (**a**, **b**) Representative bright-field image, surface area, and forming efficiency of SI-derived organoids. (**a**) Treatment with 0.5 mM acetic, propionic, or butyric acids. (**b**) Treatment with acetic and propionic acids in the absence or presence of Gpr41/43 antagonists. Scale bars: 50 μm (**a**); 100 μm **(b**). Statistical analyses were performed by one-way ANOVA with post hoc Tukey's test (**a**) and two-tailed paired t-test (**b**). n=3. *$p < 0.05$, **$p < 0.01$, ***$p < 0.001$.

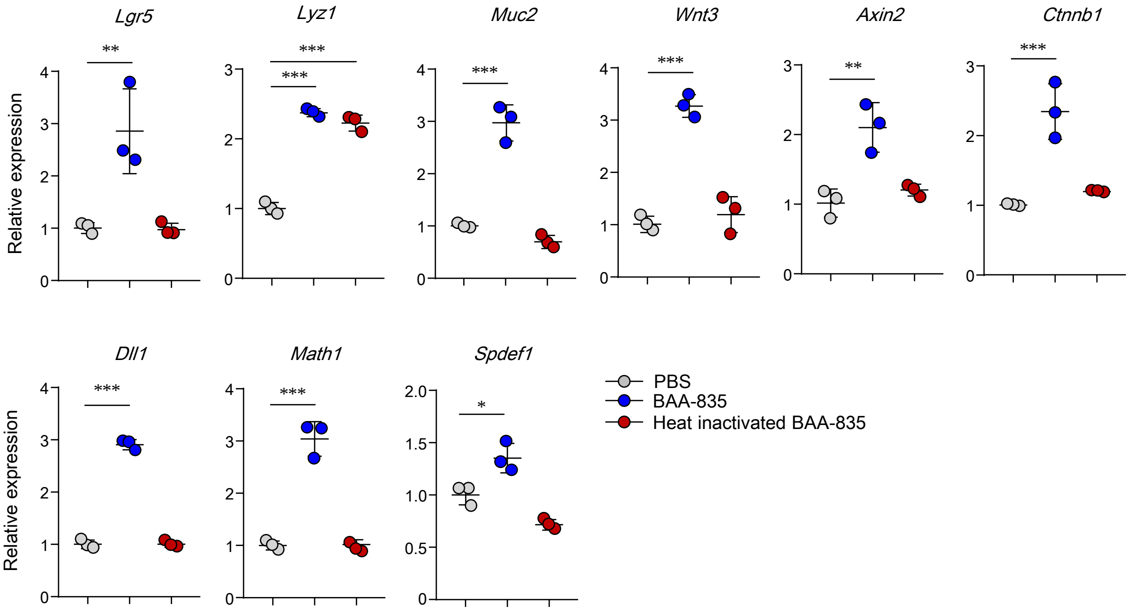

Figure S4. Effectiveness of pasteurized *A. muciniphila* BAA-835 on ISC-mediated epithelial development. Mice treated with heat-inactivated BAA-835 strain (8 x 10^8 CFU per dose) every day for four weeks. mRNA levels of *Lgr5*, *Lyz1*, *Muc2*, *Wnt3*, *Axin2*, *Ctnnb1*, *Dll1*, *Math1*, and *Spdef1* were addressed in SI tissues. Statistical analyses were carried out by one-way ANOVA with post hoc Tukey's test. n=3. *$p < 0.05$, **$p < 0.01$, ***$p < 0.001$.

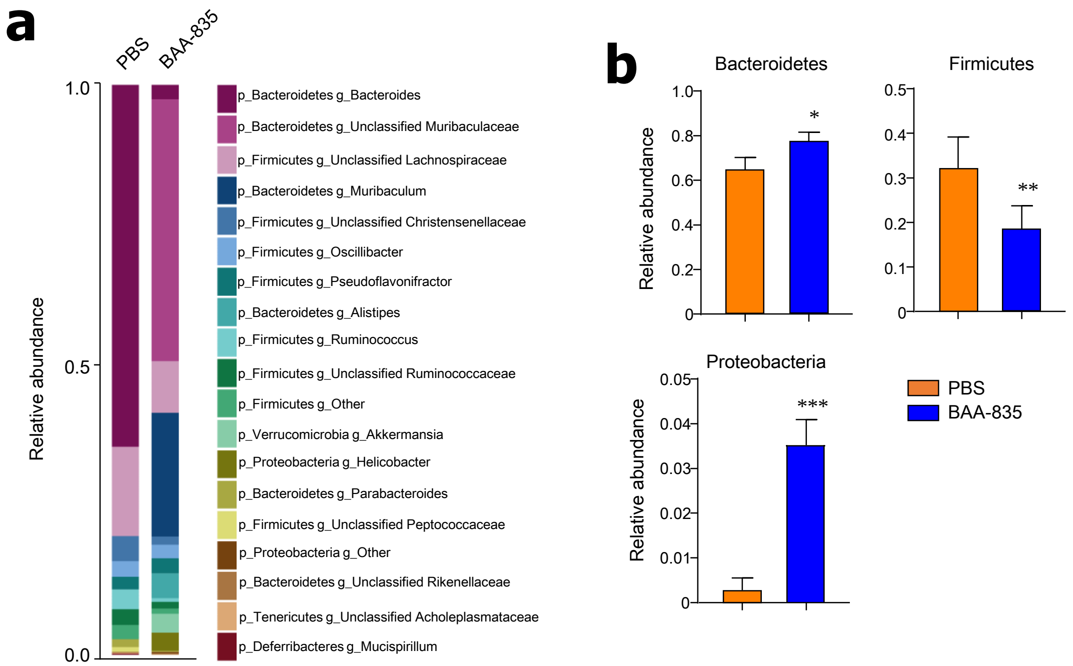

Figure S5. Microbiota composition in feces from *A. muciniphila* BAA-835-treated mice. (**a**) Fecal microbiota composition at the genus level. (**b**) Relative abundance of the phyla, Bacteroidetes, Firmicutes, and Proteobacteria. Statistical analyses were performed by two-tailed paired t-test. n=4. $^{*}p < 0.05$, $^{**}p < 0.01$, $^{***}p < 0.001$. Data were combined from ≥2 independent experiments.

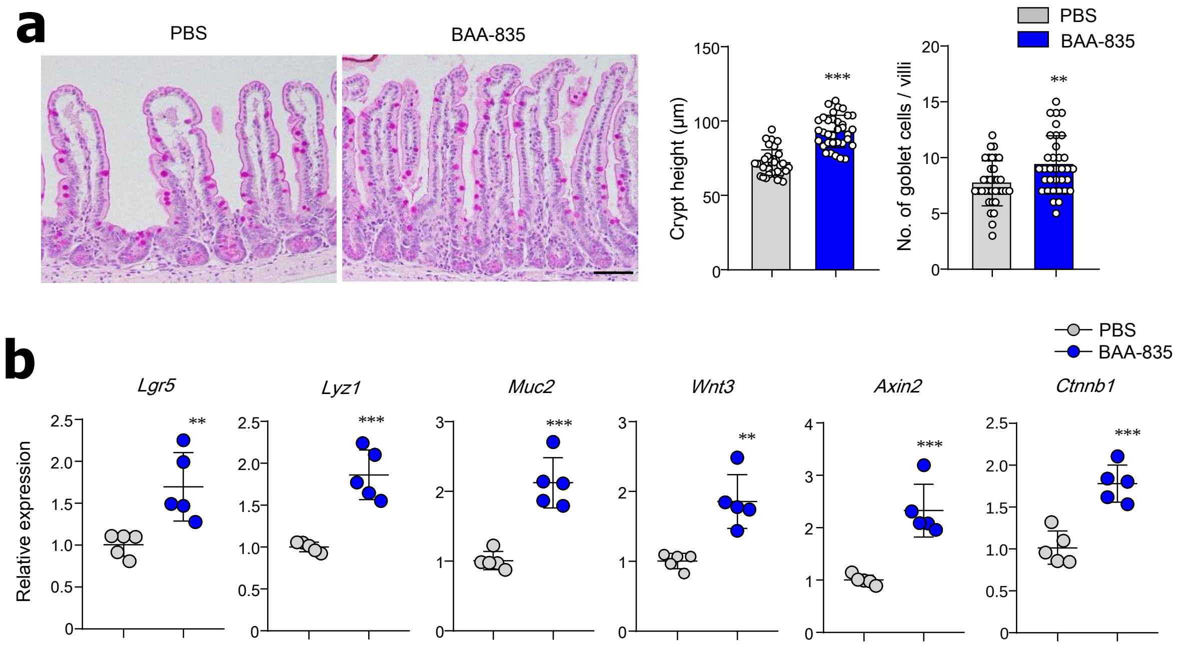

Figure S6. Effectiveness of *A. muciniphila* BAA-835 in the germ-free mice. Germ-free mice treated with BAA-835 had enhanced ISC-mediated epithelial development. (**a**) PAS staining of SI and quantification of goblet cell numbers and crypt heights. (**b**) mRNA levels of *Lgr5, Lyz1, Muc2, Wnt3, Axin2,* and *Ctnnb1* in SI tissues. Scale bars: 50 μm (**a**). Statistical analyses were carried out by two-tailed paired t-test. n=5. *$p < 0.05$, **$p < 0.01$, ***$p < 0.001$.

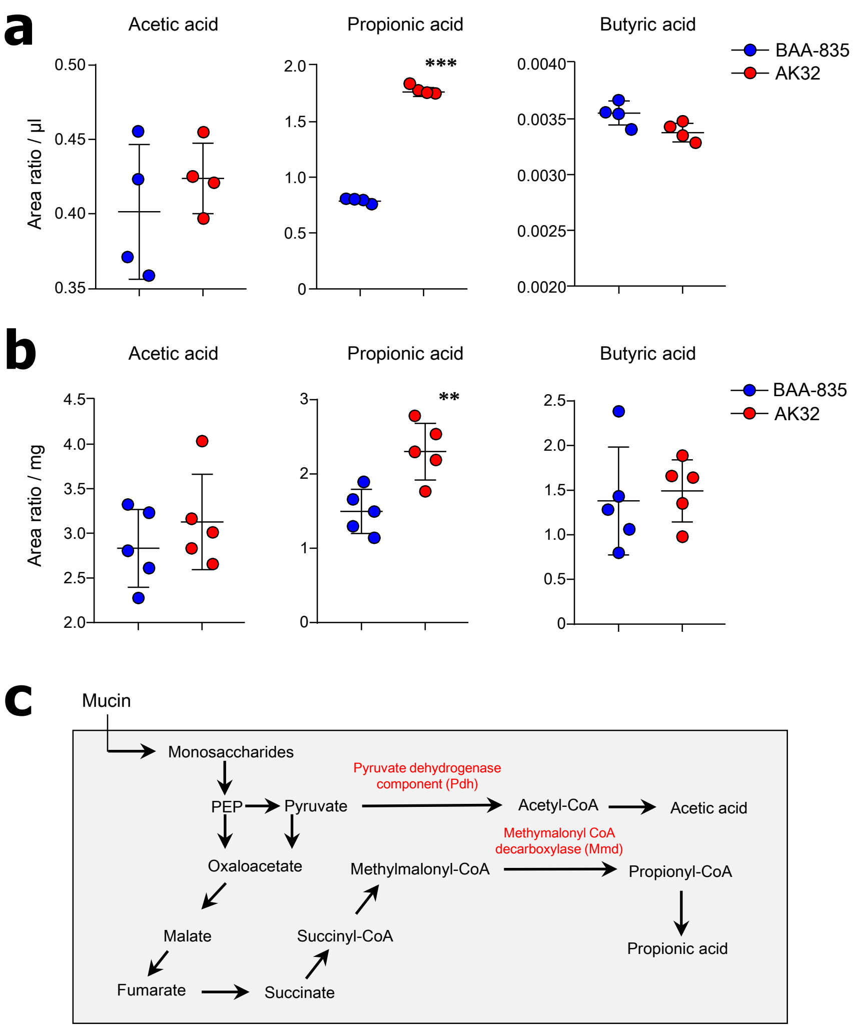

Figure S7. Quantification of SCFA concentration and metabolite production pathway. (**a**) Quantification of acetic, propionic, and butyric acids in culture supernatants of *A. muciniphila* BAA-835 or AK32 strain. (**b**) Quantification of acetic, propionic, and butyric acids in cecal contents of mice treated with BAA-835 or AK32 strains. (**c**) Model of metabolic pathway for production of acetic and propionic acids. Statistical analyses were performed by two-tailed paired t-test. $^{**}p < 0.01$, $^{***}p < 0.001$.

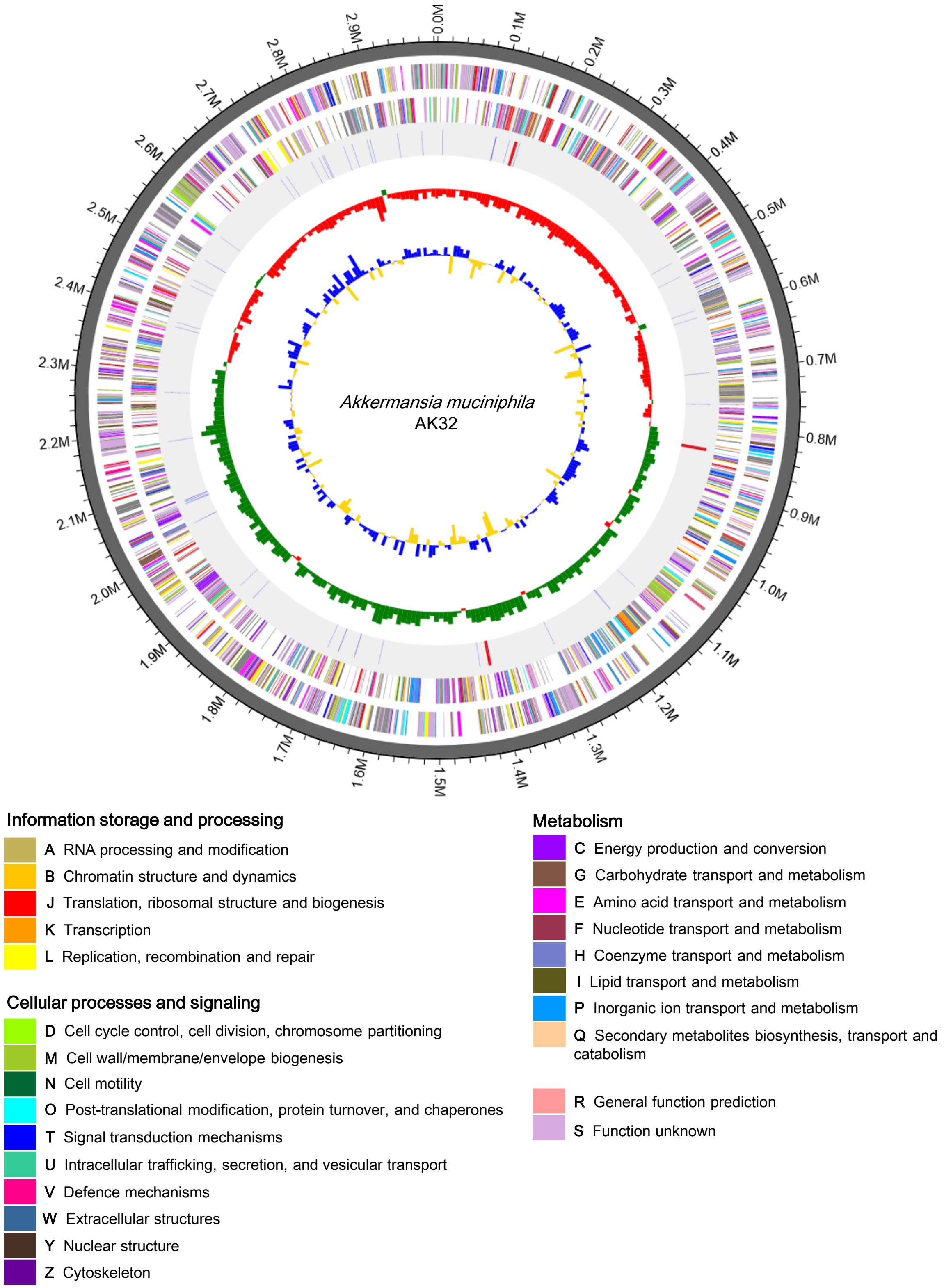

Figure S8. Complete genome map of *A. muciniphila* AK32 strain. All coding sequences were categorized by Clusters of Orthologous Groups (COG) functional categories and colored differently.

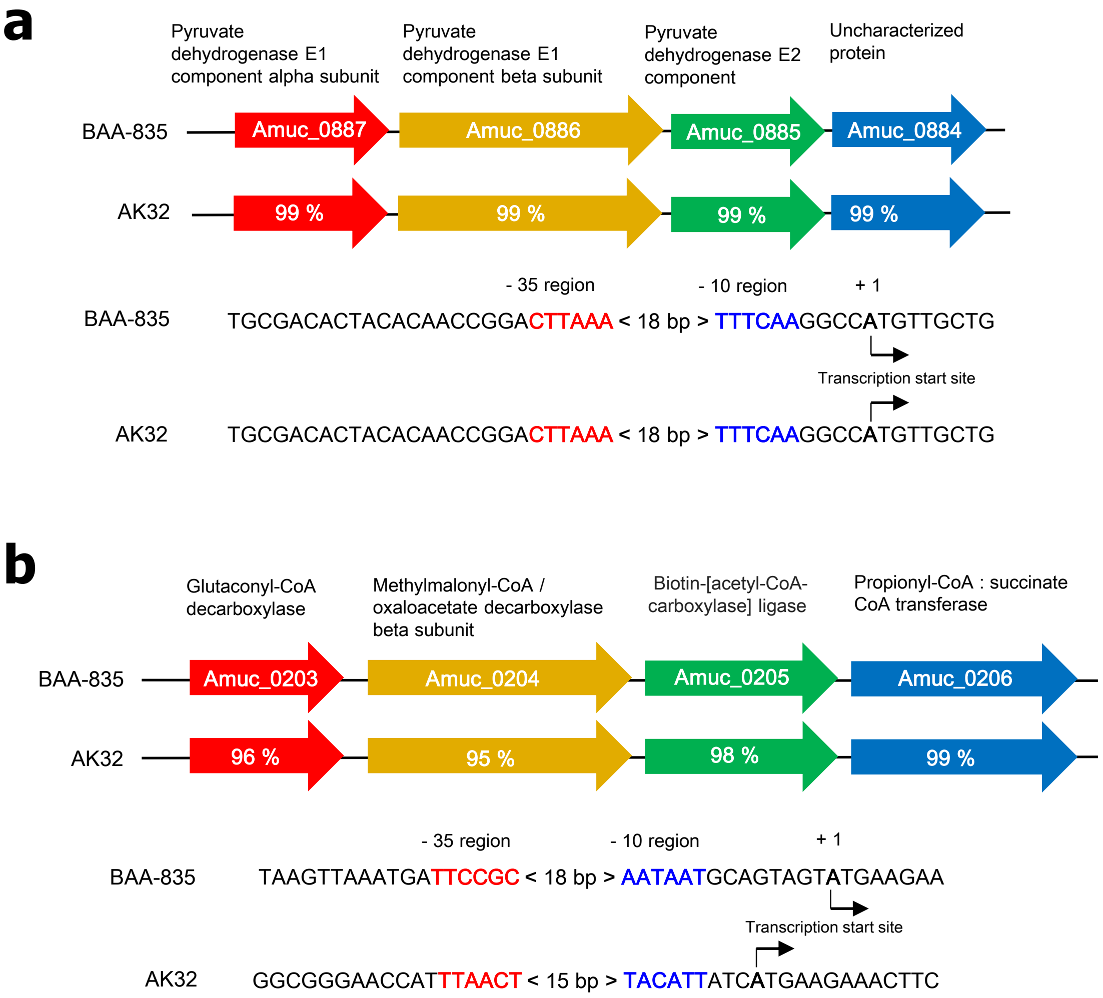

Figure S9. Homogeneity of *pdh* and *mmd* between *A. muciniphila* BAA-835 and AK32 strains. (**a**) Operon-containing genes for production of acetic acid and related promoter sequences. (**b**) Operon-containing gene for propionic acid and related promoter sequences.

a

```
BAA-835  MLLARAFDTKISSLYKAGKITGGVYLGRGHEAIAACGGVFLTAGYDVIAPFIREQAARVT
AK32     MLLARAFDTKISSLYKAGKITGGVYLGRGHEAIAACGGVFLTAGYDVIAPFIREQAARVT
         ************************************************************

BAA-835  WGEPIIEAARAYLGSALGYMKGRDGNVHRGLPAEGYMAPISHLGSTVAFVIGCLFAKRLD
AK32     WGEPIIEAARAYLGSALGYMKGRDGNVHRGLPAEGYMAPISHLGSTVAFVIGCLFAKRLD
         ************************************************************

BAA-835  GKLPGPVGVAFCGDGTTSTGAFHEAANMANVERLPLVLVVTNNQFAYSTPNIREFGEASL
AK32     GKLPGPVGVAFCGDGTTSTGAFHEAANMANVERLPLVLVVTNNQFAYSTPNIREFGEASL
         ************************************************************

BAA-835  ADRGRGYGFTVHETDGTDFMATLETFRTAVNNAREGRGPQWVLAKTLRMCGHGEHDDASY
AK32     ADRGRGYGFTVHETDGTDFMATLETFRTAVNNAREGRGPQWVLAKTLRMCGHGEHDDASY
         ************************************************************

BAA-835  IPRELKEEYEKKDPVAVAERQLLAAGWLTPEETAALKKQYADEVQLAVATAQREPEPDPF
AK32     IPRELKEEYEKKDPVAVAERQLLAAGWLTPEETAALKKQYADEVQLAVATAQREPEPDPF
         ************************************************************

BAA-835  REDWNATVWRPY
AK32     REDWNATVWRPY
         ************
```

b

```
BAA-835  MSLFDSLITFLQGMGVFSLSWQMVGMWGIAILLLYLGVAKQYEPLLMVPIAFGALIANIP
AK32     MSLFDSLITFLQGMGVFSLSWQMVGMWGIAILLLYLGVAKQYEPLLMVPIAFGALIANIP
         ************************************************************

BAA-835  DNGMLITQLNQQVISSNEQGEVTATSLNNVGYLRVHVAPLQQAPSKVPANLTTPESRAQY
AK32     DNGMLITQLNQQVISSNEQGEVTSTSLNNVGYLRVHVAPLQQTPAKVPANLTTPEARAQY
         ***********************:******************:*:**********:****

BAA-835  QEIMQQPMQVYPGSQLTVSKIKSVRESQEKAKADAARLGDDSLTVDPNLKDFQNVTEDNG
AK32     LETMQQPMQVYPGSQLTVSKIKSVRESQEKAKADAARLGDDSLTVDPNLKDFQNVTEDNG
          * *********************************************************

BAA-835  NEPVFLLTNGEGTTVVRQQGVNYFDTSGNRIPVDLKTQKLEPLVVSAAGKYVAVGQHTQE
AK32     NEPVFLLTNGEGTTVVRQQGVNYFDTSGNRVPVDLKTQKLEPLVVSAAGKYVAVGQHTQE
         ******************************:*****************************

BAA-835  LLVTSIHGGLYDWIGLGIKAEIFPPIIFLGVGALTDFGPLLAAPRTLLLGAAAQVGVAAT
AK32     LLVTSIHGGLYDWIGLGIKAEIFPPIIFLGVGALTDFGPLLAAPRTLLLGAAAQVGVAAT
         ************************************************************

BAA-835  FFMALFMGFTPNESASIGIIGGADGPTSIFLTMKLAPHLLGAVAVAAYTYMSLVPLIQPP
AK32     FFMALFMGFNPNEAASIGIIGGADGPTSIFLTMKLAPHLLGAVAVAAYTYMSLVPLIQPP
         *********.***:**********************************************

BAA-835  IMALLTTKKERLIRMKSLRTVSKSEKLFFAVLVTIVTILLIPDASPLIGMLMLGNFLREC
AK32     IMALLTTKKERLIRMKSLRTVSKSEKLFFAVLVTIVTILLIPDASPLIGMLMLGNFLREC
         ************************************************************

BAA-835  KVTERLVQASQNEIINIVTIFLGTSVGLTMQGDRFLQSETLLIILLGIVAFGVATAGGVI
AK32     KVTERLVQASQNEIINIVTIFLGTSVGLTMQGDRFLQAETLLIILLGIVAFGVATAGGVI
         *************************************:**********************

BAA-835  AAKIMNLIWRKNPVNPLIGSAGVSAVPMAARVSHNVGQKYDPSNYLLMHAMGPNVAGVIG
AK32     AAKLMNLIWRKNPVNPLIGSAGVSAVPMAARVSHNVGQKYDPSNYLLMHAMGPNVAGVIG
         ***:********************************************************

BAA-835  TAVIAGYYIATLAK
AK32     TAVIAGYYIATLAK
         **************
```

Figure S10. Alignment of Pdh (a) and Mmd (b) amino acid sequences of *A. muciniphila* BAA-835 and AK32 strains.

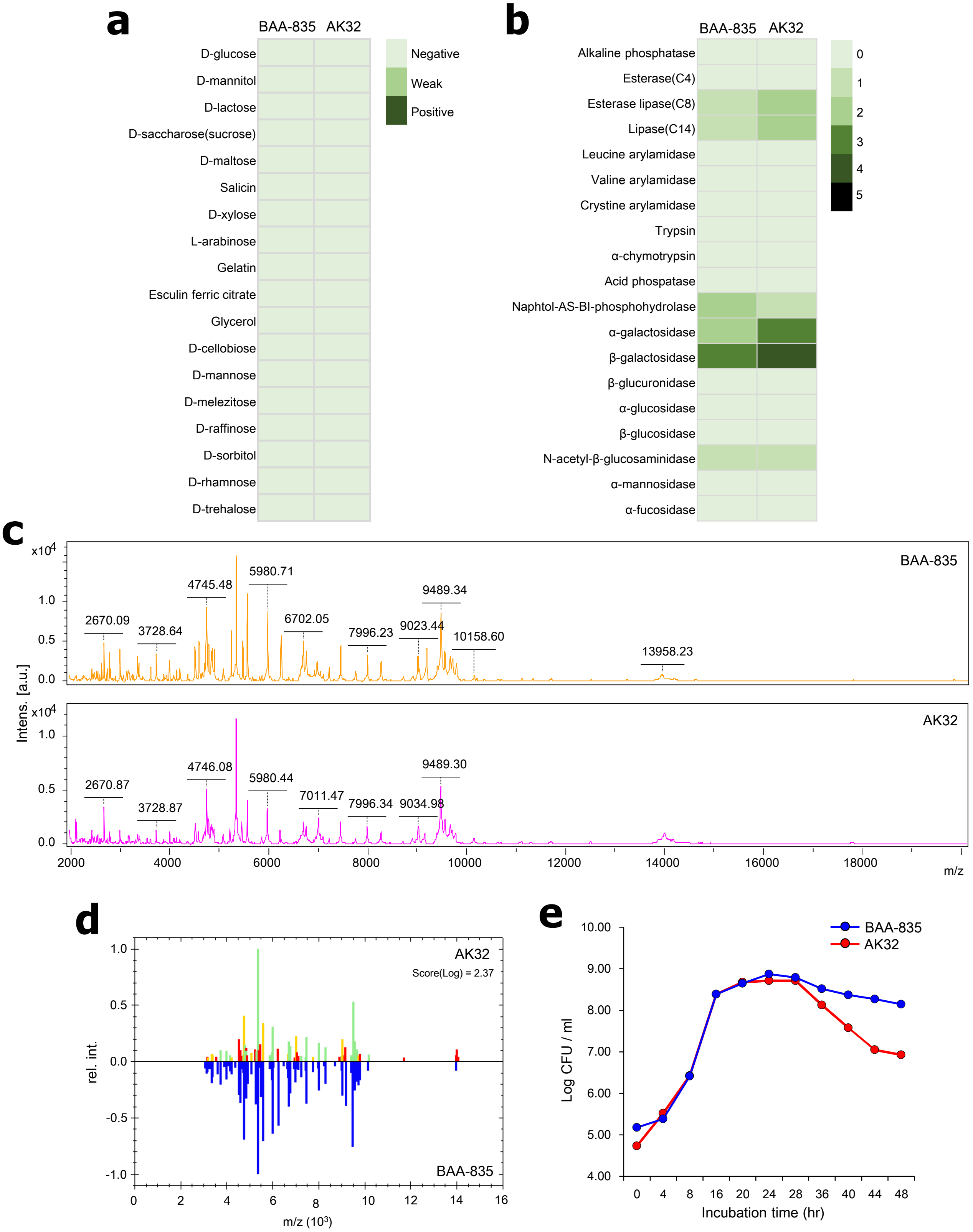

Figure S11. Characterization of *A. muciniphila* to determine a novel strain. Profiling of carbohydrate fermentation (**a**) and enzyme activity (**b**) of BAA-835 and AK32 strains. (**c**) MALDI-TOF MS spectral profiles of *A. muciniphila*. Intens. [a.u]: Intensity [arbitrary unit]. (**d**) Matching spectrum of AK32 against BAA-835. Green: Peak match within experimental error; Yellow: Close peak match, but not within experimental error; Red: No matching peak. (**e**) Comparison study for growth of BAA-835 and AK32 strains.

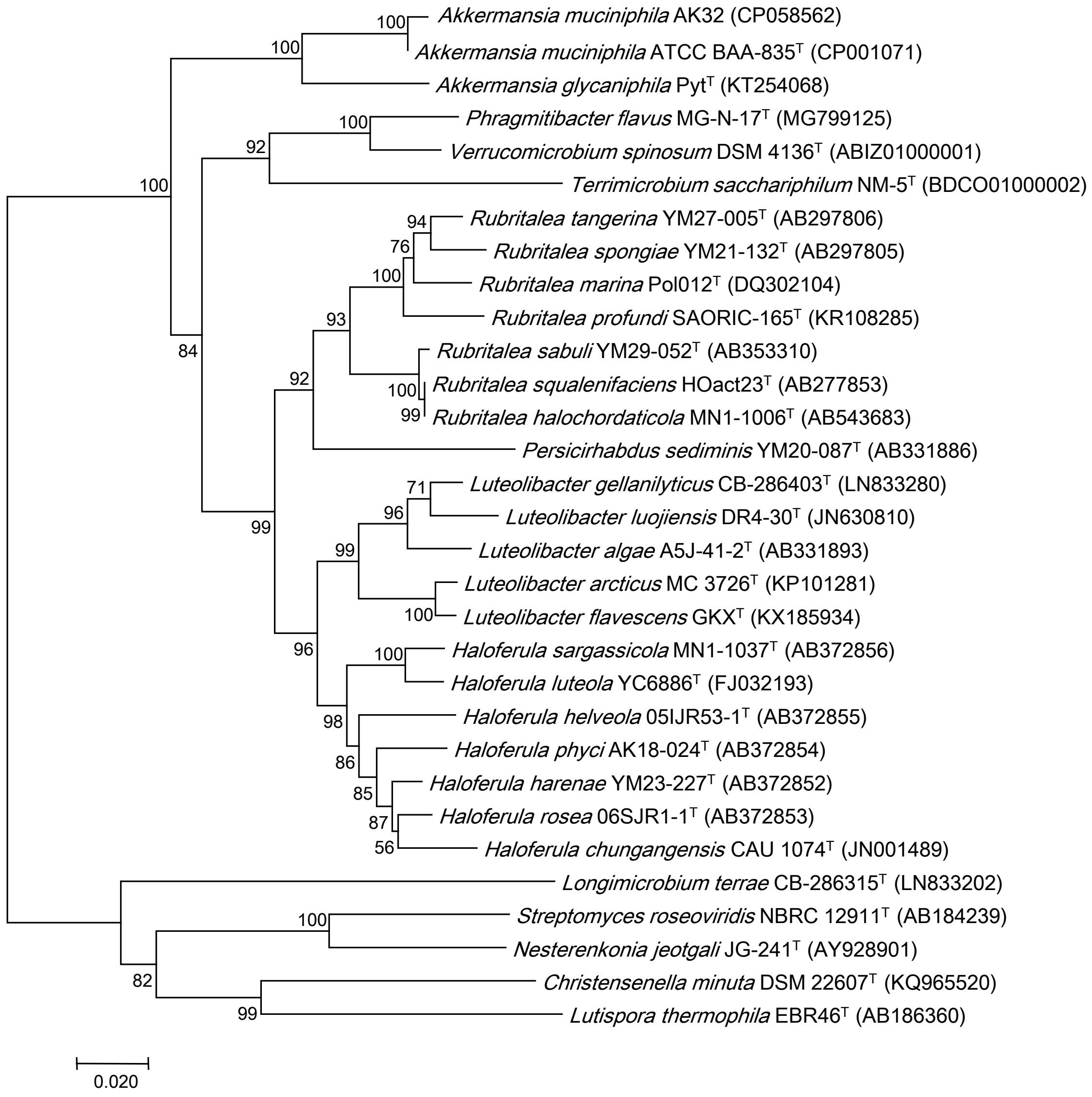

Figure S12. Phylogenetic tree of *A. muciniphila* and related taxa. Phylogenetic tree was inferred using 16S rDNA sequences.

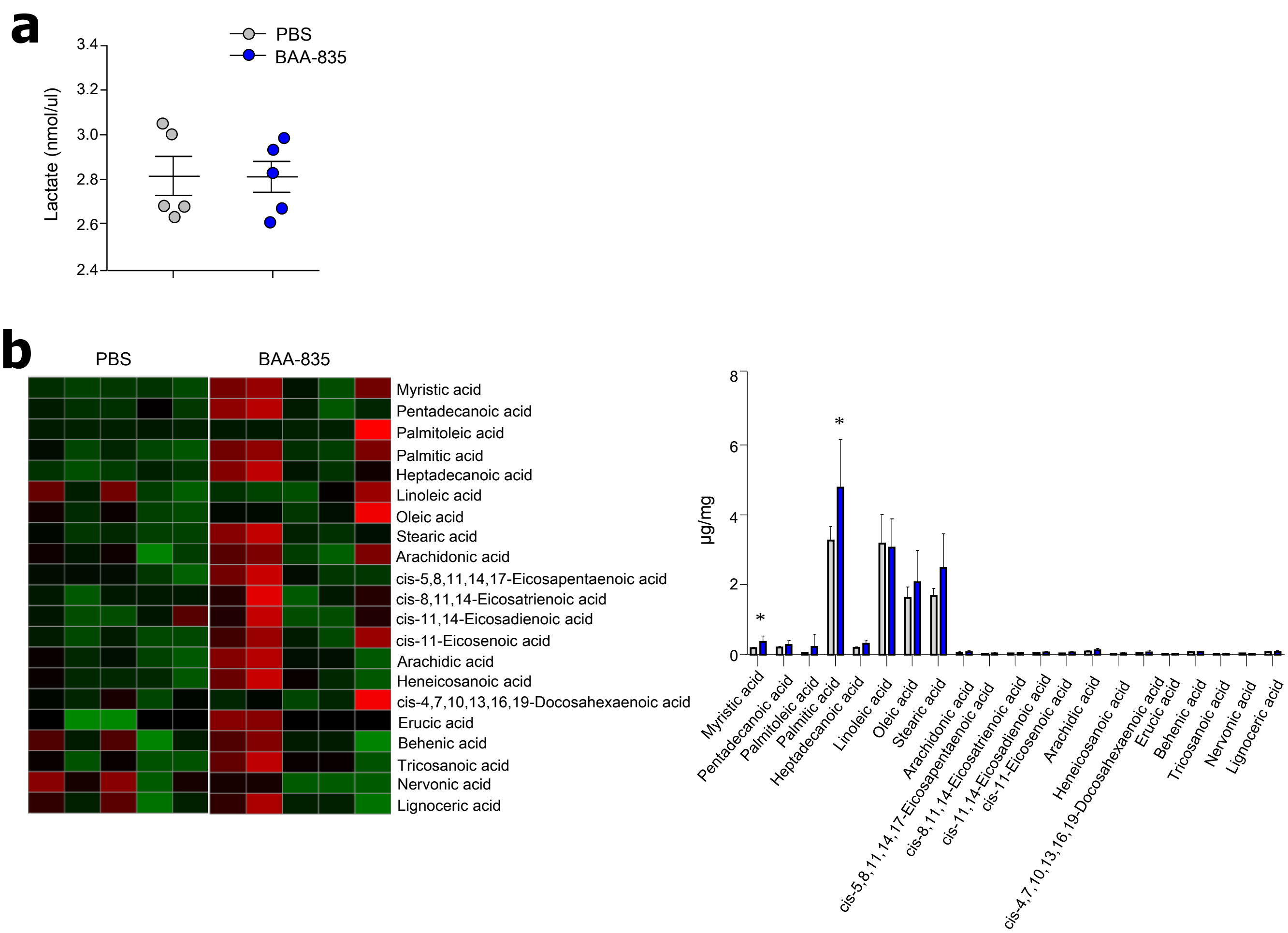

Figure S13. Quantification of lactic acid and free fatty acids in cecal contents of mice treated with *A. muciniphila* BAA-835. **(a)** Lactic acid levels in cecal content. (**b**) Heat map and quantification of free fatty acids derived from cecal content. Statistical analyses were performed by two-tailed paired t-test. n=5. $^{*}p < 0.05$.

Table S1. Summary of trimming information.

	Pre-filter	Post-filter
Polymerase Read Bases	1,038,559,582	990,444,044
Polymerase Reads	150,292	95,828

Table S2. Summary of genome annotation of ATCC BAA-835 and AK32 strains.

Strain	Base (bp)	No. of CDS*	GC contents (%)	tRNA	rRNA	ANIb† (%)
BAA-835	2,664,102	2,184	55.8	53	9	-
AK32	3,004,919	2,600	55.3	56	9	97.39

* Coding sequences

† Average nucleotide identity based BLAST to type strain ATCC BAA-835
